# PRMT3 Drives IDO1-Dependent Radioresistance and Immunosuppression by Promoting Kynurenine Metabolism in Non–Small Cell Lung Cancer

**DOI:** 10.1158/0008-5472.CAN-24-4162

**Published:** 2025-10-23

**Authors:** Shijie Zhang, Siyu Wang, Yixue Wang, Tianle Zhou, Jiaxin Yang, Jingxue Xu, Gen Li, Yunyan Zhang, Xiaoyuan Wang, Hang Yin

**Affiliations:** 1 Department of Radiation Therapy, Harbin Medical University Cancer Hospital, Harbin, China.; 2 Department of Thoracic Surgery, Harbin Medical University Cancer Hospital, Harbin, China.; 3 Key Laboratory of Tumor Immunology in Heilongjiang, Harbin Medical University Cancer Hospital, Harbin, China.; 4 The Hong Kong Polytechnic University, Hong Kong, China.

## Abstract

Radioresistance is one of the primary causes of treatment failure in non–small cell lung cancer. In this study, we identified protein arginine methyltransferase-3 (PRMT3) within tumors as a critical mediator impairing both radiotherapy sensitivity and T-cell antitumor activity. In tumor cells, PRMT3 mediated arginine methylation of transcription factor TFAP2A, enhancing its binding to the indoleamine 2,3-dioxygenase 1 (IDO1) promoter. Methylated TFAP2A exhibited a prolonged half-life, increased nuclear localization, and enhanced dimer formation, which ultimately led to heightened IDO1 expression that enhanced synthesis of kynurenine (Kyn). The elevated Kyn production boosted radioresistance in tumor cells and suppressed the infiltration and function of CD8^+^ T cells by activating aryl hydrocarbon receptors. Interestingly, combined pharmacologic inhibition of PRMT3 and IDO1 effectively disrupted the TFAP2A–IDO1–Kyn axis, overcoming radioresistance, activating antitumor immunity, and ultimately eradicating tumors. In conclusion, these findings delineate PRMT3-mediated Kyn metabolism as a mechanism of radioresistance and immune evasion in non–small cell lung cancer, offering valuable insights for potential interventions for treating patients with lung cancer.

**Significance:**

PRMT3 orchestrates metabolic reprogramming in non–small cell lung cancer through a TFAP2A-IDO1 pathway that stimulates kynurenine synthesis to promote radioresistance and immunosuppression, highlighting this axis as a putative therapeutic target.

## Graphical Abstract

**Figure:**
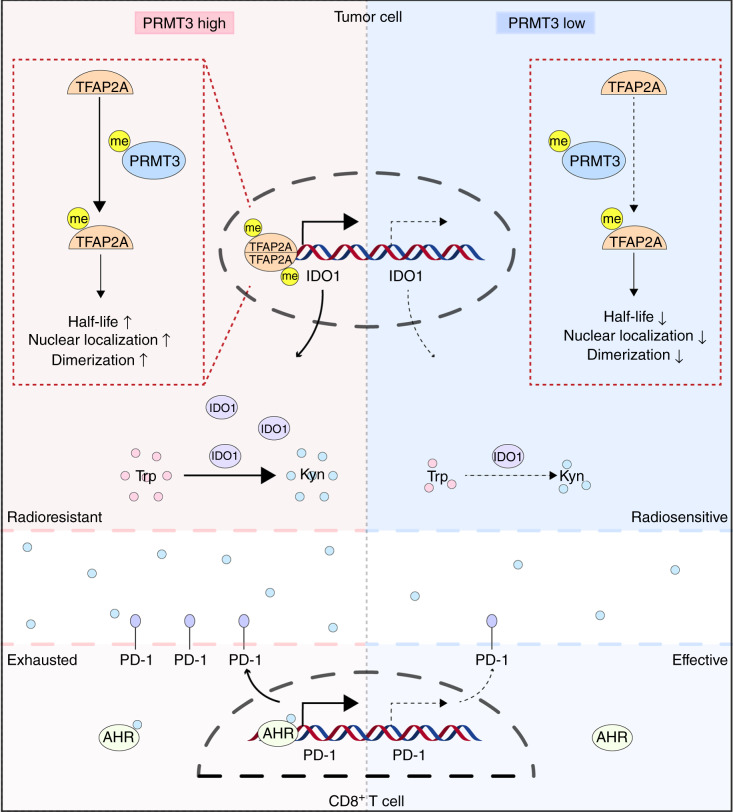

## Introduction

Lung cancer has emerged as a widespread public health concern (1). Non–small cell lung cancer (NSCLC) represents the primary type of cancer, exerting a profound influence on global human health (2). Radiotherapy resistance contributes to the recurrence and metastasis of NSCLC after treatment, thereby reducing patient survival. The N^6^-methyladenosine modification of RNA (3), transcription factors (4), and macrophage polarization (5) are regarded as pivotal factors in modulating cancer radiosensitivity. Specific gene expression profiles might underlie tumor resistance to radiotherapy (6). Further investigation into tumor gene expression patterns and regulatory networks associated with radiotherapy represents an effective approach to addressing treatment resistance.

Recent studies have shown that the metabolic state of tumors evolves during disease progression (7, 8). Determining how metabolic reprogramming supports tumor progression and identifying relevant reprogramming activities could lead to effective treatments for tumor recurrence. The kynurenine (Kyn) metabolic pathway primarily handles the breakdown of tryptophan (Trp) in the human body (9). Indoleamine 2,3-dioxygenase 1 (IDO1) catalyzes the degradation of Trp and the synthesis of Kyn (10). Tumors exhibit elevated Kyn metabolism, with IDO1 frequently overexpressed in various cancers, and its expression level is inversely related to patients’ overall survival time (11). In NSCLC, more than half of the patients exhibit IDO1 positivity (10), and its overexpression is linked to chemotherapy resistance (12). High IDO1 activity in patients prior to radiotherapy correlates with a greater risk of radiation-induced lung injury (13). Increased IDO1 activity after radiotherapy is associated with worse survival outcomes in patients with stage III NSCLC undergoing concurrent or sequential chemoradiotherapy (14). Furthermore, IDO1 and PD-L1 are coexpressed in cases of NSCLC with radiotherapy (15). Suppression of IDO1 can overcome resistance to anti–PD-1 therapy (16). By catalyzing the synthesis of Kyn, IDO1 fosters an immunosuppressive microenvironment, characterized by the suppression of effector CD8^+^ T cells (17, 18). However, the failure of several clinical trials for inhibitors aimed at blocking IDO1 activity and Kyn metabolism indicates the importance of patient stratification for treatments based on IDO1 inhibitors, and the complexity of regulating upstream Kyn metabolism with tissue-specific effects may be associated with tumors (19, 20). Elucidating the interactions between IDO1 and other pathways, as well as the mechanisms behind the constitutive activation of IDO1 in cancer, is crucial for understanding other pathways that may compensate for IDO1 function and developing effective treatment strategies (21–23).

Protein arginine methylation is a kind of extensive posttranslational modification mediated by the protein arginine methyltransferase (PRMT) family, which plays an important role in a variety of pathophysiologic processes and diseases. On the one hand, PRMT can regulate tumor cell death (24), mitochondrial homeostasis (25), and fatty acid synthesis (26) to mediate tumor progression and therapeutic response. On the other hand, arginine methylation is also the hub of activating the IFN pathway and tumor immunity (27–29). Nevertheless, further exploration is needed on the role of PRMT in lung cancer radiotherapy resistance.

In this study, we identified the tumor-intrinsic PRMT, PRMT3, as a significant player in the resistance to radiotherapy of NSCLC, regulating Kyn metabolism via the TFAP2A–IDO1 axis within tumor cells. The enhanced release of Kyn into the tumor microenvironment (TME) activates aryl hydrocarbon receptors (AhR) in CD8^+^ T cells, promoting immune evasion by inducing T-cell exhaustion. Importantly, our research demonstrates that targeting PRMT3 can modulate the infiltration and function of CD8^+^ T cells. Pharmacologic inhibition of PRMT3 bolsters the efficacy of radiotherapy in suppressing tumor growth, thereby presenting a potential therapeutic strategy for NSCLC.

## Materials and Methods

### Cohorts of patients with NSCLC

We collected information and tumor tissue sections from 113 patients diagnosed with NSCLC at Harbin Medical University Affiliated Cancer Hospital. Tumor treatment response was assessed 1 month after radical radiotherapy according to RECIST version 1.1. Complete response (CR) means that total target lesions disappear and no new lesions appear. Partial response (PR) refers to a reduction of at least 30% in the sum of the diameters of target lesions from baseline, with CR not being achieved. Progressive disease (PD) is characterized by an increase of at least 20% in the sum of the diameters of target lesions or the appearance of new lesions. Stable disease is a state between PD and PR, indicating no significant change in the size of target lesions. Patients with CR or PR are defined as responders, whereas those with stable disease or PD are considered nonresponders. All target lesions were evaluated on CT scans (layer thickness ≤5 mm). The study followed the ethical principles of the Helsinki Declaration. The study was approved by the Ethics Committee of Harbin Medical University Affiliated Cancer Hospital, and all patients signed written informed consent forms.

### IHC and hematoxylin and eosin staining

In the realm of IHC staining, the research involving human tissues in this investigation received clearance from the Ethical Review Board at Harbin Medical University, with each participant providing their consent via a signed form. The specimens were preserved in formalin and embedded in paraffin, followed by the preparation of 4-μm-thick tissue sections. After the process of deparaffinization and rehydration, the slides underwent treatment with a solution designed for antigen retrieval. Subsequently, the slides were soaked in hydrogen peroxide (H_2_O_2_) and a solution containing goat serum. The primary antibody was then applied to the slides and allowed to bind at a temperature of 4°C throughout the night. Following this, the slides were exposed to a secondary antibody that was biotinylated. The antibodies used in the experiment are listed in Supplementary Table S1.

The IHC score was calculated by multiplying the staining intensity score by the proportion of positive cells score. The intensity of staining was categorized as follows: negative (0), weak (1), moderate (2), and strong (3). The proportion of positive cells was scored as follows: no positive cells (0), ≤25% (1), 26% to 50% (2), 51% to 75% (3), and >75% (4). This assessment was independently conducted by two experienced pathologists who were blinded to the clinical outcomes. The gene expression levels were categorized into high and low expression groups based on the median IHC score as the cutoff.

For hematoxylin and eosin staining, tissue sections were dewaxed and rehydrated to remove paraffin and restore the tissue to a hydrated state. The sections were then stained with hematoxylin to color the nuclei and subsequently stained with eosin to highlight the cytoplasm.

### qRT-PCR

The total RNA was extracted from the specimens utilizing the TRIzol RNA extraction reagent (Invitrogen). The process of converting RNA to cDNA and subsequent PCR amplification was conducted using a reverse transcription kit (TOYOBO). Primers and SYBR (TOYOBO) were added to amplify with the StepOnePlus RT-PCR System. GAPDH served as a benchmark for normalization, and the data were calculated using the 2^−ΔΔC_t_^ method to assess the relative expression levels. The primers utilized were supplied by Seven Biotech, and the sequences are published in Supplementary Table S2. The protocols followed were in accordance with those of previous research and adhered to the manufacturer’s guidelines.

### Western blotting

The cells were lysed on ice with a solution of RIPA supplemented with 1 mmol/L phenylmethylsulfonyl fluoride (PMSF), and the concentration of the extracted proteins was determined using a bicinchoninic acid assay kit. Proteins were resolved using SDS-PAGE, and subsequently, they were transferred onto polyvinylidene difluoride membranes (Millipore; 200–250 mA; 60–140 minutes). After being blocked with a solution containing 5% skim milk, the membranes were exposed to the corresponding primary antibodies with incubation at 4°C throughout one night. On the subsequent day, the membranes were further incubated with the respective secondary antibodies at ambient temperature. Ultimately, the chemiluminescent signal was captured at the antibody–protein-binding sites on the membrane using an enhanced chemiluminescence detection reagent, and the luminescent image was recorded with a Tanon 5200 imaging system.

NSCLC tissues frozen in liquid nitrogen were cut into small pieces and ground. RIPA containing PMSF was added. The cells were treated with 20 μmol/L cycloheximide (Sigma-Aldrich, C7698) at different times to detect the half-life of protein. For the detection of dimer, add 2 mmol/L disuccinimidyl suberate (Aladdin, D155694) to the PBS-cleaned cells, cross-linked at room temperature for 30 minutes, add the termination solution Tris-HCl (BOSTER, AR1163) until the final concentration of Tris is 10 to 20 mmol/L, and then lyse the cells after 15 minutes of termination at room temperature. The antibodies pertinent to the experiment are presented in Supplementary Table S1.

### Cell lines and cell culture

Human NSCLC cell lines H1299, H460, H1993, H1915, and A549 were sourced from the Institute of Cell Biology under the Chinese Academy of Sciences. The mouse Lewis lung cancer (LLC) cell line was purchased from Procell. Regular authentication of these cell lines was conducted through short tandem repeat DNA profiling, ensuring the absence of *Mycoplasma* contamination. The human cells were maintained in a culture medium composed of RPMI-1640 (Gibco), supplemented with 10% FBS (ScienCell) and 1% penicillin–streptomycin solution (Beyotime, C0222). Mouse cell line was cultivated in DMEM (Gibco). The cells were incubated under conditions of 37°C in an atmosphere containing 5% CO_2_.

### Cell transfection

By using lentiviral vector, PRMT3 (oePRMT3) or matched control vector (oeNC) was stably overexpressed in human NSCLC cell line and mouse cell lines. PRMT3 knockout (shPRMT3) or control (shNC) transfection in human cell lines were also achieved by lentivirus. After infection, the cell lines were treated with puromycin for a period of 2 weeks to select for cells with stable expression, which was then confirmed through Western blot. Additionally, GeneChem was responsible for the construction of plasmids aimed at targeting IDO1 (shIDO1 and oeIDO1), TFAP2A (shTFAP2A and oeTFAP2A), and mutants, along with a negative control for comparison. The target sequences for short hairpin RNA are listed in Supplementary Table S3.

### Clonogenic survival assay

Exponentially growing cells at 70% to 80% confluence from each group were seeded into six-well plates at different densities (400 cells/well for 0 Gy, 600 cells/well for 2 Gy, 800 cells/well for 4 Gy, 1200 cells/well for 6 Gy, and 2,400 cells/well for 8 Gy). Irradiation was performed 12 hours later. The cells were cultured for 14 days and then fixed with 4% paraformaldehyde (PFA) solution and stained with 0.5% crystal violet solution. Colonies consisting of >50 cells were counted. The surviving fraction (SF) and plating efficiency (PE) were calculated using the following formulas:

SF = (Colonies formed after irradiation)/[(Cells seeded before irradiation) × PE]

PE = (Colonies formed for control)/(Cells seeded for control)

The clonogenic survival curve is generated by fitting the relationship between the SF and the radiation dose using the linear–quadratic equation (L-Q model), expressed as follows: y = exp (−αx − βx^2^).

### Wound-healing assay

The cells were seeded into a six-well plate. After the cells grew to 100% density, a scratch was formed on the plate with the tip of a 200 μL pipette, and then the cells were washed with PBS. Then the cells were kept in serum-free medium, and plate images were taken at 0-hour and 24-hour intervals under a microscope. The experiment was repeated three times.

### Transwell

NSCLC cells were cultured into the 24-well Transwell plates with 8.0-μm pores (Corning Costar). The top compartment of the apparatus was filled with 200 μL of medium devoid of serum, which housed 1 × 10^5^ cells, whereas the bottom compartment received 600 μL of medium enriched with 10% FBS. Following a 24-hour incubation period at a temperature of 37°C, the compartments were rinsed with PBS, fixed with a 4% solution of PFA for a duration of 20 minutes, and subsequently stained with a 0.4% solution of crystal violet.

### EdU

Meilun EdU Cell Proliferation Kit with Alexa Fluor 555 (Meilunbio, MA0425-1) was used to detect the proliferation of cells. The cells to be tested were seeded into six-well plates, cultured overnight until they returned to normal state, and then added to EdU. The cells were cultured in a packed box for 1 hour. After EdU labeling, the culture medium was removed and fixed at room temperature for 15 to 30 minutes by adding fixed solution. Wash the cells three times, add 1 mL permeating solution, and incubate at room temperature for 10 to 15 minutes. Remove the detergent from the previous step. A measure of 500 μL click reaction solution was added to each well, and the cells were incubated at room temperature without light for 30 minutes. After washing the cells for three times, 1 × Hoechst 33342 solution was added, and the cells were incubated at room temperature without light for 10 minutes. Use a fluorescent microscope to photograph.

### Reactive oxygen species assay

To ascertain the levels of reactive oxygen species (ROS) within the cells, they were first rinsed with PBS and then treated with dihydroethidium (Solarbio, CA1420) at a temperature of 37°C for a duration of 30 minutes. After staining, the cells underwent two rounds of washing with PBS. Subsequently, the cells were visualized using a fluorescence microscope (LEICA). The fluorescence intensity before and after stimulation was measured at an excitation wavelength of 518 nm and an emission wavelength of 610 nm.

### Organoids

The corresponding experiment was conducted according to the instructions of the human NSCLC organoid culture medium kit (K2 ONCOLOGY, KNSCLC-100). NSCLC tissue samples were collected and stored in tissue preservation solution. Surgical scissors were used to remove mixed fat or muscle tissue. After full cleaning, the tissue was cut into small pieces <1 mm^3^. Enzymolysis with tissue enzyme at 37°C was performed for 30 to 60 minutes. 5% FBS stops digestion. Add 2 mL of red blood cell (RBC) lysis solution and incubate at room temperature for 3 to 5 minutes to lyse RBCs. After centrifugation, the supernatant was removed, and the cells were resuspended with organ-like basic medium. After centrifugation, the supernatant was sucked and discarded, and the cell precipitation was suspended with matrix glue, dropped on the bottom of the 24-well cell culture plate, and cultured in a 37°C, 5% CO_2_ incubator.

### Patient-derived tumor xenograft model experiments

The tumor tissue obtained from patients with NSCLC by surgical resection was collected and put into tissue preservation solution and cut into small pieces. These tumor fragments were then implanted under the skin of the right hind limb of NKG mice ages 6 to 8 weeks. After three consecutive rounds of tumor proliferation in mice, the tumor was evaluated by IHC to ensure that the tumor retained its original histologic characteristics. It was further implanted into the right abdominal region of NKG mice.

Starting from the 10th day after establishing the patient-derived tumor xenograft (PDX) model, each mouse subgroup received intraperitoneal injections of 10 mg/kg PRMT3 inhibitor (SGC707, HY-19715) once every 2 days until the end of the study. Tumors were irradiated with 6 MeV electron ray at a dose rate of 3.5 Gy/minute, delivering 8 Gy once daily for three consecutive days using a Clinac CX linear accelerator (Varian Medical Systems). During this period, closely monitor indicators and provide regular updates on tumor growth and mouse weight fluctuations. Use an electronic caliper to determine the tumor size and estimate the volume by multiplying the formula [length (L) × width squared (W^2^)] by 0.52. After the experiment, tissue samples were collected and analyzed using IHC and flow cytometry. The animal experiment plan has been approved by the Animal Ethics Committee of Harbin Medical University Cancer Hospital.

### Coimmunoprecipitation

Coimmunoprecipitation (co-IP) assays were performed using a co-IP kit (abs955, absin). The cells were lysed with the lysate from the co-IP kit added with PMSF to obtain the required protein samples. Add 5 μL protein A and 5 μL protein G, incubate at 4°C for 30 to 60 minutes, and retain the supernatant after centrifugation. The corresponding IP primary antibody or IgG antibody was added and incubated overnight at 4°C. Add 5 μL protein A and 5 μL protein G and incubate overnight at 4°C. After centrifugation, add 1 × SDS sample buffer, and heat the sample to 95°C to 100°C for 5 minutes. The samples were analyzed by Western blotting.

### 4D-fast DIA qualitative proteomics

In order to identify PRMT3-interacting proteins, protein samples were obtained by co-IP. The sample was removed from −80°C and melted on ice. An appropriate amount of triethylammonium bicarbonate (TEAB) was added to adjust pH 8.0. Five μL of suspension was used for SDS-PAGE testing. For digestion, the protein solution was reduced with 5 mmol/L dithiothreitol for 30 minutes at 56°C and alkylated with 11 mmol/L iodoacetamide for 15 minutes at room temperature in darkness. The protein sample was then diluted by adding 200 mmol/L TEAB to urea concentration less than 2 mol/L. Finally, trypsin was added at 1:50 trypsin-to-protein mass ratio for the first digestion overnight and 1:100 trypsin-to-protein mass ratio for a second 4-hour digestion. Peptides were analyzed by mass spectrometry (MS; PTM BIO). Finally, the peptides were desalted by Strata-X Solid-Phase Extraction (SPE) column. The resulting MS/MS data were processed using DIA-NN search engine (v.1.8).

### Untargeted metabolomics

The samples were placed in the microcentrifuge (EP) tubes and resuspended with prechilled 80% methanol by well vortex. Then the samples were melted on ice and whirled for 30 seconds. After the sonification for 6 minutes, they were centrifuged at 5,000 rpm, 4°C for 1 minute. The supernatant was freeze-dried and dissolved with 10% methanol. Ultra-high-performance liquid chromatography (UHPLC)-MS/MS analysis was conducted by GeneChem. The raw data files generated by UHPLC-MS/MS were processed using the Compound Discoverer 3.3 (CD3.3, Thermo Fisher Scientific) to perform peak alignment, peak picking, and quantitation for each metabolite.

### ELISA

In order to detect the contents of Trp and Kyn, ELISA was performed using human Trp kits (MEIMIAN, MM-51157H1) and human Kyn kits (MEIMIAN, MM-51191H1) according to the manufacturer’s instructions. Trp and Kyn in mouse tumors and coculture systems were measured using mouse Trp kits (MEIMIAN, MM-0756M1) and mouse Kyn kits (MEIMIAN, MM-0755M1).

### RNA sequencing

The total RNA was extracted from the specimens utilizing the TRIzol RNA extraction reagent (Invitrogen). Sequencing libraries were created following the manufacturer’s instructions using the NEBNext UltraTM RNA Library Prep Kit for Illumina (NEB). Unique index codes were added to each sample for identification. In short, mRNA was extracted from total RNA using poly-T oligo-attached magnetic beads. The mRNA was then fragmented at an elevated temperature with divalent cations in NEBNext First-Strand Synthesis Reaction Buffer (5×). First-strand cDNA synthesis was performed using random hexamer primers and reverse transcriptase, followed by second-strand cDNA synthesis with buffer, dNTPs, DNA polymerase I, and RNase H. The library fragments were purified using the QIAquick PCR Kit, eluted with EB buffer, and then underwent terminal repair, A-tailing, and adapter ligation. The desired library products were then purified and PCR-amplified to complete library preparation. The RNA concentration of the prepared library was measured using the Qubit RNA Assay Kit on the Qubit 3.0 system and diluted to 1 ng/µL. Library insert size was evaluated with the Agilent Bioanalyzer 2100 system (Agilent Technologies) and accurately quantified to have an insert size and concentration of more than 10 nmol/L using the StepOnePlusTM Real-Time PCR System. For clustering, index-coded samples were processed on a cBot cluster generation system using the TruSeq PE Cluster Kit v3-cBot-HS (Illumina) according to the manufacturer’s guidelines. Following cluster generation, libraries were sequenced on an Illumina NovaSeq platform, producing 150 bp paired-end reads.

### Isolation and detection of T cells

According to the manufacturer’s instructions, EasySep Mouse CD8a Positive Selection Kit II (STEMCELL Technologies, 18953) was used to isolate CD8^+^ T cells from mouse spleen. The cells were cultured in RPMI-1640 supplemented with 10% FBS and 1 ng/mL IL2 (Abbkine, PRP157) and activated with anti-CD3 antibody (Thermo Fisher Scientific, 16-0031-81) and anti-CD28 antibody (Thermo Fisher Scientific, 16-0281-81). Lung cancer cells were seeded into 12-well plates and cocultured with activated CD8^+^ T cells at a ratio of 1:2 for 48 hours. The cells were collected for CD45, CD3, CD8, granzyme B, and propidium iodide staining. The samples were analyzed by flow cytometry. The antibodies used are listed in the Supplementary Table S4.

### Dual-luciferase reporter assay

The empty pcDNA3.1, TFAP2A-pcDNA3.1 plasmid and the target gene IDO1 promoter wild-type/mutant plasmid were purchased from Gene Create (Supplementary Table S5). H1993 and H1299 cells were seeded in 24-well plates at a density of 1 × 10^5^ cells/well and cultured for 24 hours. Then, the plasmid was transfected with Polyplus jetPRIME. After incubation for 48 hours, luciferase activity was detected in the luminometer according to the manufacturer’s instructions of the double-luciferase reporter gene detection kit (Gene Create, JKR23008).

### Chromatin immunoprecipitation–qPCR

The chromatin immunoprecipitation (ChIP) assay was performed utilizing the SimpleChIP Plus Sonication Chromatin IP Kit (Cell Signaling Technology, 9003). Initially, NSCLC cells were cross-linked with formaldehyde (1% final concentration) for 10 minutes, followed by a 5-minute incubation with glycine at room temperature. The cells were then collected and resuspended in cell lysis and nuclear lysis buffers, and the chromatin was fragmented using the ultrasonicator. Following fragmentation, the chromatin was incubated overnight at 4°C with rotation using anti-TFAP2A (1:100) or IgG (1 μg). The final DNA was then purified and analyzed by qPCR. The ChIP–qPCR primer for the IDO1 promoter is provided in Supplementary Table S2.

### Immunofluorescence and multiplex immunofluorescence

Cells that were cultivated on confocal dishes were stabilized with a solution containing 4% PFA and rendered permeable with 0.1% Triton X-100. For the preparation of the seal, 3% BSA was introduced into the dish, to which the diluted antibody aimed at the target antigen was added for the incubation process, and this setup was left in a humidified environment overnight. On the subsequent day, the cells were processed with a fluorescent secondary antibody that matched the primary, for a period of 1 hour at room temperature. The nuclei of the cells were subsequently delineated by staining with DAPI. For fluorescence colocalization analysis, use FlexAble logo Antibody Labeling Kits (Proteintech, KFA002 and KFA003) to label the antibody before adding the primary antibody and restain DAPI without the need for secondary antibody incubation. Imaging was performed under LSM880 with Airyscan confocal microscope (Zeiss). After euthanasia of mice, tumors were extracted and stored overnight in 4% PFA. After paraffin embedding, the four-color multiplex fluorescence IHC staining kit (Absin, abs50028) was used to stain tumor sections from mice. The relevant antibodies are listed in Supplementary Table S6.

### Xenograft tumor model

Four-to-six week old female BALB/c nude mice and C57BL/6 mice were assigned to our study and injected with 5 × 10^6^ LLC cells. When the average volume of the tumor reaches 80 to 100 mm^3^, the following treatments should be performed. For the subgroup that requires treatment with IDO1 inhibitor 1-methyl-Trp (1-MT; Sigma, 452483), drinking water with a drug concentration of 2 mg/mL was given immediately after tumor formation, and sucrose was added to increase the acceptance of mice, ensuring that each mouse drank 4 to 5 mL of water per day. SGC707 (MedChemExpress, HY-19715) was also administered intraperitoneally at a dose of 10 mg/kg once every 2 days after tumor formation. Two days after tumor formation, mice were given tumor radiotherapy, 8 Gy once a day for three consecutive days. InVivoPlus anti-mouse CD8α (Bio X Cell, BP0061), 350 μg/mouse/time, intraperitoneal injection, once every 4 days. Anti-mouse PD-L1 (B7-H1)-InVivo (Selleck, A2115), 10 mg/kg, intraperitoneal injection, once every 3 days. Measure tumor volume every 3 days.

### Flow cytometry analysis

The obtained mouse tumors were washed three times in PBS, and collagenase IV was added to digest at 37°C until the liquid was turbid. The tumor mass was ground and sieved in a 70-μm cell sieve. Add RBC lysate to lyse for 5 minutes, twice. The obtained cell clusters were washed with PBS containing 2% FBS. The corresponding antibodies were incubated at 4°C in dark for staining. The apoptosis detection kit (Meilunbio, MA0220) was used to detect cell apoptosis. LSRFortessa analytic flow cytometer was used for sample analysis, and Flowjo software was used for data analysis.

### Molecular docking

Using PRMT3 and TFAP2A as keywords, retrieve protein structures from UniProt and screen for human species in the database. Set appropriate docking parameters and use HDOCK for protein–protein docking. The results include two indicators, one is the docking score, in which a more negative docking score indicates a more likely combination model; the second is the confidence score. Confidence_score = 1.0/[1.0 + e^0.02^ × (docking_score + 150)]. When the confidence score is higher than 0.7, the likelihood of two molecules binding is high; when the confidence score is between 0.5 and 0.7, it is considered that two molecules can bind; when the confidence score is below 0.5, it is considered that the likelihood of two protein molecules binding is low.

### Bioinformatic analysis

Download and organize RNA sequencing (RNA-seq) data and clinical data of The Cancer Genome Atlas (TCGA)-lung adenocarcinoma (LUAD) and TCGA-lung squamous cell carcinoma (LUSC) from the TCGA database. Differential expression analysis of two conditions/groups (two biological replicates per condition) was performed using the DESeq2 R package (1.16.1). DESeq2 provide statistical routines for determining differential expression in digital gene expression data using a model based on the negative binomial distribution. The resulting *P* values were adjusted using the Benjamini–Hochberg approach for controlling the FDR. Genes with an adjusted *P* < 0.05 found by DESeq2 were assigned as differentially expressed. Prior to differential gene expression analysis, for each sequenced library, the read counts were adjusted by edgeR program package through one scaling normalization factor. Differential expression analysis of two conditions was performed using the edgeR R package (3.18.1). The *P* values were adjusted using the Benjamini–Hochberg method. Corrected *P* value of 0.05 and absolute fold change of 2 were set as the threshold for significantly differential expression. Based on the single-sample gene set enrichment analysis algorithm provided in R package gene set variation analysis (GSVA; 1.46.0), the immune infiltration status of the corresponding cloud data is calculated using the markers of 24 immune cells provided in the Immunity article (30). Perform Spearman correlation analysis using ggplot2 (3.3.6).

### Statistical analyses

Statistical differences between two groups were assessed using a two-tailed unpaired or paired Student *t* test, as appropriate. For comparisons involving multiple groups, one-way ANOVA was performed. Correlations between groups were analyzed using Spearman correlation test. Survival analysis was conducted using the Kaplan–Meier method. Data are presented as the mean ± SD or SEM, as indicated. All statistical analyses were performed using GraphPad Prism 8.0 software. In general, all experiments were repeated at least three independent times. A *P* value of less than 0.05 was considered to indicate statistical significance.

## Results

### PRMT3 facilitates the resistance to radiotherapy in NSCLC

**Figure 1.**
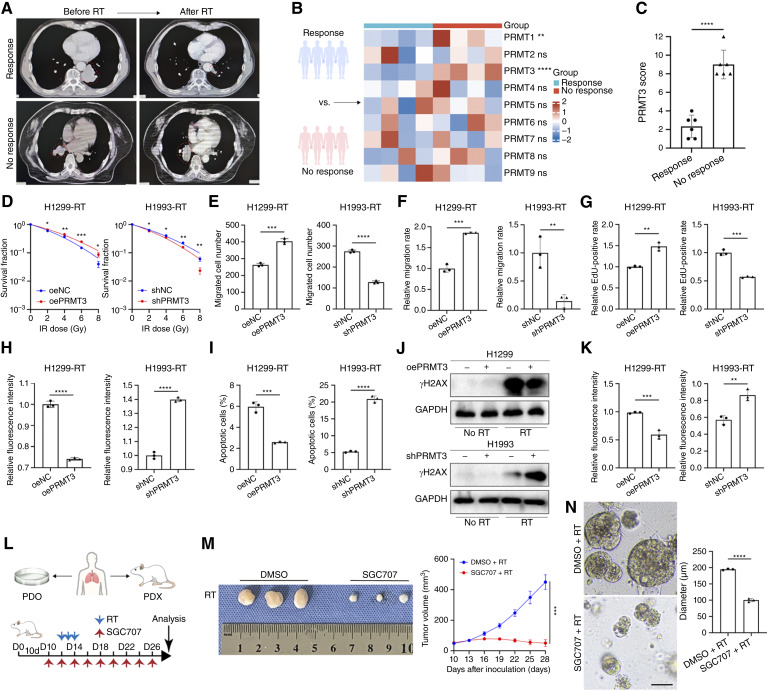
PRMT3 promotes radiotherapy resistance in NSCLC. **A,** Tumor response to radiotherapy (RT) was classified into response and nonresponse groups based on changes in tumor size after treatment. **B,** The mRNA expression levels of nine PRMT family genes were quantified in both response and nonresponse groups using RT-PCR, with GAPDH serving as a loading control (*n* = 4). NSCLC tissues were collected from patients prior to any treatment. **C,** PRMT3 expression was significantly elevated in the nonresponse group (*n* = 6). **D,** Overexpression of PRMT3 enhanced the clonogenic survival of NSCLC cells after radiotherapy (0–8 Gy), whereas PRMT3 knockout reduced clonogenic survival (*n* = 3). **E–G,** Overexpression of PRMT3 promoted the migration and proliferation of NSCLC cells after radiotherapy (4 Gy; *n* = 3). **H,** Overexpression of PRMT3 inhibited the production of ROS in cells after radiotherapy (4 Gy; *n* = 3). **I,** Knockdown of PRMT3 promoted apoptosis in NSCLC cells 24 hours after radiotherapy (6 Gy; *n* = 3). **J,** NSCLC cells from the designated group were subjected to 6 Gy irradiation and subsequently cultured for 1 hour under standard conditions, followed by Western blot analysis using γH2AX antibodies. **K,** IF confocal microscopy was used to examine the regulatory effect of PRMT3 expression on γH2AX formation after radiotherapy (6 Gy; *n* = 3). **L,** PDX tumors were treated with the PRMT3 inhibitor SGC707 (10 mg/kg, every 2 days via intraperitoneal injection) and/or radiotherapy (8 Gy/day, three consecutive days). D, day; PDO, patient-derived tumor organoid. **M,** Tumor growth curve was plotted (*n* = 3 mice per group). **N,** Corresponding patient-derived tumor organoids were treated with SGC707 (10 μmol/L) and/or radiotherapy (8 Gy, once). Scale bar, 100 μm. Data represent the mean ± SD. Differences were tested using two-way ANOVA test (**B**) and unpaired two-sided Student *t* test (**C–I**, **K**, **M**, and **N**). ns, not significant; *, *P* < 0.05; **, *P* < 0.01; ***, *P* < 0.001; ****, *P* < 0.0001.

To identify the specific PRMT that plays a pivotal role in radiation resistance, we collected a patient cohort with NSCLC (Supplementary Table S7). After radiotherapy, the groups with and without response were defined based on tumor changes (Fig. 1A). qRT-PCR detection showed that patients in the radiotherapy response group had low expression of PRMT1 and PRMT3, with the most significant difference in PRMT3 expression between the two groups (Fig. 1B). Therefore, we speculate that PRMT3 may be a key factor in regulating the radiotherapy sensitivity of NSCLC. IHC analysis of tumor slices before treatment showed that the expression level of PRMT3 was higher in the radiotherapy nonresponsive group (Fig. 1C; Supplementary Fig. S1A). The same results were obtained by Western blot (Supplementary Fig. S1B). To elucidate the effect of radiotherapy on the gene expression profile of NSCLC cells, we analyzed RNA-seq data of cells before and after radiotherapy in GSE25814. The results showed that PRMT3 was upregulated after radiotherapy (Supplementary Fig. S1C; Supplementary Table S8). We conducted a detailed analysis of PRMT3 expression in NSCLC and normal tissues within the TCGA database, along with its influence on the survival rates of patients with NSCLC. The ROC curve of the diagnostic subjects indicated that PRMT3 has the best AUC value among the nine PRMT family members (Supplementary Fig. S1D). The differential expression analysis of the LUAD combined with LUSC datasets from the TCGA database indicates that PRMT3 exhibits higher expression in tumor tissues than in the normal tissues (Supplementary Fig. S1E). Survival analysis showed that high expression of PRMT3 was associated with low overall survival rate (Supplementary Fig. S1F). The above results indicate that PRMT3 may be an important factor in regulating the radiosensitivity of NSCLC.

We delved deeper into the interplay between PRMT3 and radiotherapy resistance. Based on their endogenous PRMT3 expression levels, we selected H1993 cells for PRMT3 knockdown and H1299 cells for PRMT3 overexpression (Supplementary Fig. S1G and S1I). In cells subjected to radiotherapy, PRMT3 expression escalated in tandem with escalating radiotherapy doses (Supplementary Fig. S1H). Clonogenic survival assays revealed that PRMT3 overexpression endowed H1299 cells with radioresistance, whereas its suppression in H1993 cells increased the susceptibility to radiation (Fig. 1D; Supplementary Fig. S2A). Elevated PRMT3 levels were associated with enhanced post-radiotherapy cell migration and proliferation (Fig. 1E–G; Supplementary Fig. S2B–S2D). Conversely, PRMT3 knockdown augmented the radiotherapy-induced ROS production (Fig. 1H; Supplementary Fig. S2E). Furthermore, we utilized flow cytometry to scrutinize the apoptotic changes. Notably, PRMT3 overexpression markedly decreased the apoptosis rate in H1299 cells, whereas its knockdown had the opposite effect in H1993 cells (Fig. 1I). Using Western blot and immunofluorescence (IF) laser confocal microscopy, we measured the levels of γH2AX. Intriguingly, PRMT3 overexpression significantly diminished the γH2AX foci count (Fig. 1J and K; Supplementary Figs. S1J and S3A). Subsequently, we established PDX models and patient-derived tumor organoid models from NSCLC patient tumor tissues (Fig. 1L; Supplementary Fig. S3B). We used SGC707 (a selective allosteric inhibitor of PRMT3) to investigate the role PRMT3 plays in NSCLC radioresistance. Treatment of PDX models and subcutaneous tumor models with SGC707 alongside radiation demonstrated a significant radiosensitization effect (Fig. 1M; Supplementary Fig. S3C and S3E). Notably, SGC707 treatment led to a reduction in Ki-67 tumor staining (Supplementary Fig. S3D). Furthermore, our findings were corroborated by organoid cultures derived from tumor tissues of newly diagnosed patients with NSCLC, further supporting the role of PRMT3 in enhancing radiotherapy resistance (Fig. 1N).

### PRMT3-mediated Kyn metabolism promotes radiotherapy resistance in NSCLC

**Figure 2.**
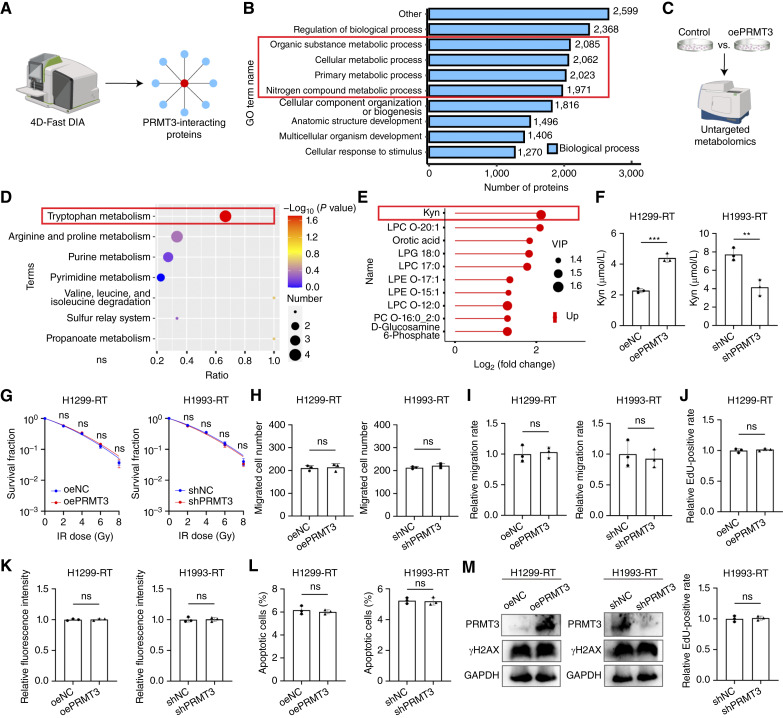
Kyn metabolism is a key mediator of PRMT3 function in NSCLC. **A,** 4D-Fast DIA qualitative proteomics was utilized to identify PRMT3-interacting proteins from co-IP experiments. **B,** Gene Ontology (GO) enrichment analysis was performed on the identified PRMT3-interacting proteins. **C,** Nontargeted metabolomics analysis was conducted on specific NSCLC cell groups. **D,** Kyoto Encyclopedia of Genes and Genomes enrichment analysis was applied to the nontargeted metabolomics data. **E,** Elevated levels of Kyn were detected in PRMT3-overexpressing cells compared with controls. Up, upregulated. **F,** The Kyn concentration in the culture supernatant of specific NSCLC cells after radiotherapy (RT; 4 Gy) was measured to assess Kyn metabolism (*n* = 3). **G–M,** The impact of PRMT3 on NSCLC cell clonogenic survival (0–8 Gy; *n* = 3; **G**), migration (4 Gy; *n* = 3; **H**), wound-healing (4 Gy; *n* = 3; **I**), and proliferation (4 Gy; *n* = 3; **J**) after radiotherapy was evaluated in Trp-depleted culture conditions. Additionally, ROS production (6 Gy; *n* = 3; **K**), apoptotic cell ratio (6 Gy; *n* = 3; **L**), and γH2AX expression (6 Gy; **M**) were also assessed in Trp-depleted NSCLC cells following radiotherapy. Data represent the mean ± SD. Differences were tested using unpaired two-sided Student *t* test (**F–L**). ns, not significant; **, *P* < 0.01; ***, *P* < 0.001.

To further investigate the specific mechanism by which PRMT3 regulates radiosensitivity, we performed a MS analysis to identify interacting proteins (Fig. 2A). The Gene Ontology enrichment analysis of these interacting proteins hinted at a potential regulatory role of PRMT3 in cellular metabolism (Fig. 2B). To elucidate the mechanism underlying PRMT3’s influence on NSCLC, a comprehensive, untargeted metabonomic analysis was conducted on cells with overexpression of PRMT3 and their controls (Fig. 2C). A significant enrichment of differential metabolites was observed within the Trp metabolic pathway, particularly following PRMT3 overexpression (Fig. 2D). Notably, Kyn emerged as the most substantially upregulated metabolite in the context of PRMT3 overexpression (Fig. 2E; Supplementary Fig. S4A). ELISA analysis confirmed that overexpression of PRMT3 significantly increases Kyn metabolism whereas PRMT3 knockdown induced the converse effect (Fig. 2F). These findings indicate that PRMT3 modulates Kyn metabolism within tumor cells. Most notably, the manipulation of PRMT3 expression in culture media devoid of Trp did not influence the radiosensitivity of NSCLC cells. Specifically, the proliferation and migration of irradiated cells were not modulated by PRMT3 levels in the absence of Trp (Fig. 2G–J; Supplementary Fig. S4B–S4E). Furthermore, in the absence of Trp, the rates of radiation-induced apoptosis, ROS production, and γH2AX formation were similarly unaffected by PRMT3 overexpression or knockdown (Fig. 2K–M; Supplementary Fig. S5A). These collective findings underscore the notion that PRMT3 exerts its impact on the radioresistance of NSCLC through the modulation of Trp metabolism.

### The effect of PRMT3 on NSCLC Kyn metabolism is dependent on IDO1

**Figure 3.**
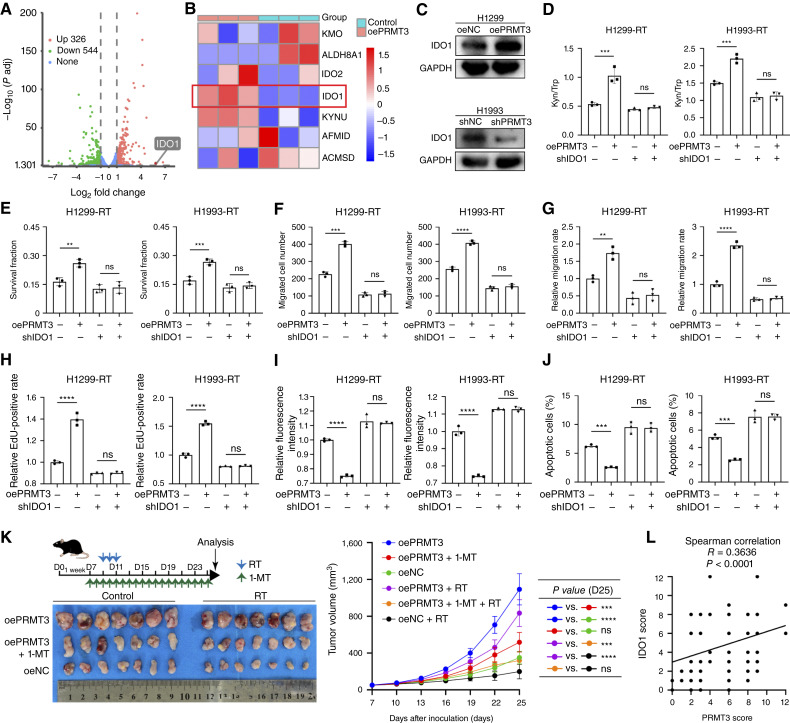
PRMT3 regulates Kyn metabolism through IDO1. **A,** RNA-seq was used to identify differentially expressed genes in PRMT3-overexpressing and control NSCLC cells. Down, downregulated; Up, upregulated. **B,** A heatmap depicting the expression of genes in the Kyn metabolism pathway. **C,** Western blot confirmed PRMT3-induced upregulation of IDO1 at protein level (*n* = 3). **D,** IDO1 knockdown reduced PRMT3-induced enhancement of Kyn metabolism in NSCLC cells after radiotherapy (RT; 4 Gy; *n* = 3). **E–J, **In parallel, IDO1 knockdown mitigated PRMT3-induced increases in clonogenic survival (6 Gy; *n* = 3; **E**), migration (4 Gy; *n* = 3; **F**), wound-healing (4 Gy; *n* = 3; **G**), and proliferation (4 Gy; *n* = 3; **H**) in NSCLC cells following radiotherapy. Additionally, IDO1 knockdown abrogated PRMT3-mediated increases in ROS production (6 Gy; *n* = 3; **I**) and apoptosis (6 Gy; *n* = 3; **J**) after radiotherapy. **K, ***In vivo*, PRMT3 promoted tumor growth in a mouse model, an effect reversed by IDO1 inhibitor (*n* = 7). D, day. **L,** A positive correlation between PRMT3 and IDO1 expression was observed in our patient cohort. Data represent the mean ± SD. Differences were tested using unpaired two-sided Student *t* test (**D–J**) and one-way ANOVA test (**K**). The correlation was determined using Spearman correlation test (**L**). ns, not significant; **, *P* < 0.01; ***, *P* < 0.001; ****, *P* < 0.0001.

To elucidate the molecular underpinnings of PRMT3’s influence on Kyn metabolism, we conducted RNA-seq to identify differentially expressed genes between PRMT3-overexpressing and control cell lines. Notably, the RNA-seq analysis revealed a significant upregulation of the gene encoding IDO1, a rate-limiting enzyme in the Kyn metabolism, in cells with elevated PRMT3 levels (Fig. 3A and B). Furthermore, Western blot and qRT-PCR assays demonstrated that PRMT3 overexpression upregulated IDO1 at both the protein and mRNA levels, whereas PRMT3 knockdown had the converse effect (Fig. 3C; Supplementary Fig. S5B). Importantly, modulation of IDO1 expression did not reciprocally affect PRMT3 levels in cells (Supplementary Fig. S5C). Furthermore, we observed that PRMT3 overexpression led to an increase in the Kyn/Trp ratio. However, when IDO1 was knocked down, PRMT3 overexpression did not result in a similar increase in the Kyn/Trp ratio (Fig. 3D). Additionally, IDO1 knockdown reduced the proliferation and invasion of PRMT3-mediated tumor cells following radiotherapy, whereas PRMT3 overexpression after IDO1 knockdown had no significant effect on cell proliferation and migration compared with cells with only IDO1 knockdown (Fig. 3E–H; Supplementary Figs. S5D–S5 and S6A). Moreover, the radiotherapy resistance induced by PRMT3 overexpression was reversed upon IDO1 knockdown, indicating that the radiotherapy-sensitizing effect of PRMT3 in NSCLC cells is dependent on IDO1 (Fig. 3I and J; Supplementary Fig. S7A and S7B).

We developed a cancer xenograft tumor model using C57BL/6 mice. In this model, overexpression of PRMT3 significantly accelerated tumor growth in mice undergoing radiotherapy compared with the control group. The administration of 1-MT, an IDO1-specific competitive protein inhibitor, effectively countered the radioresistance-enhancing effect of PRMT3 overexpression (Fig. 3K; Supplementary Fig. S7C). IHC analysis also revealed that IDO1 expression in the tumors of mice overexpressing PRMT3 was significantly elevated. The PRMT3-overexpressing group exhibiting elevated Ki-67 levels, indicative of increased cell proliferation (Supplementary Fig. S7D). In PDX model mice, tumor IDO1 expression in the PRMT3 inhibitor and radiotherapy groups was found to be lower than in the DMSO and radiotherapy groups (Supplementary Fig. S7E). Analysis of tumor tissue sections from patients with NSCLC (Fig. 3L; Supplementary Fig. S8A; Supplementary Table S9) and mouse models (Supplementary Fig. S8B) demonstrated a significant positive correlation between PRMT3 and IDO1 expression levels. These findings underscore the potential therapeutic value of targeting the PRMT3–IDO1 axis in enhancing the efficacy of radiotherapy for lung cancer.

### PRMT3-activated Kyn metabolism mediates dual effects on tumor cells and the immune microenvironment

**Figure 4.**
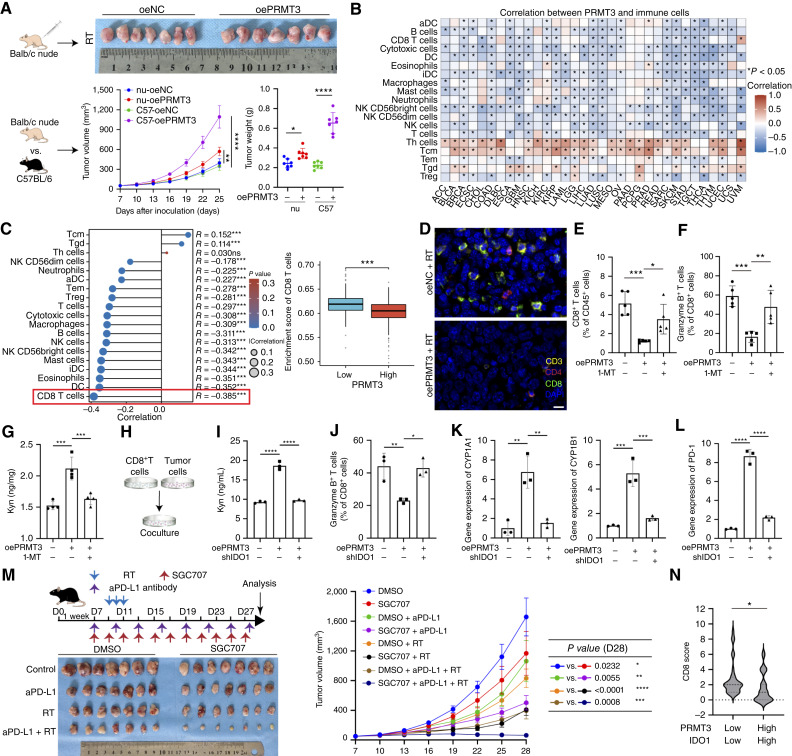
PRMT3 influences NSCLC by modulating the tumor-immune microenvironment. **A,** A subcutaneous xenograft model was constructed in immunodeficient nude mice. Compared with immune-competent mice, PRMT3 overexpression did not exhibit the same tumor-promoting effects in the nude mice (*n* = 7). RT, radiotherapy. **B,** Pan-cancer analysis from TCGA revealed that PRMT3 affects the tumor-immune microenvironment. **C,** High PRMT3 expression in patients with NSCLC was associated with reduced CD8^+^ T-cell infiltration. **D,** IF confirmed PRMT3’s inhibitory effect on CD8^+^ T cells. Scale bar, 20 μm. **E** and **F,** Flow cytometry analysis of mouse tumors was performed (*n* = 5). **G,** ELISA demonstrated that PRMT3’s effect on Kyn metabolism could be rescued by 1-MT (*n* = 3). **H–J,** Coculturing CD8^+^ T cells extracted from mouse spleen with LLC cells (**H**) showed that PRMT3-overexpressing tumor cells upregulated Kyn metabolism (**I**) and reduced granzyme B expression in CD8^+^ T cells (**J**). **K** and **L,** qRT-PCR analysis of CYP1A1, CYP1B1 (**K**), and PD-1 (**L**) expression in CD8^+^ T cells (*n* = 3). **M,** PRMT3 inhibitor enhanced the efficacy of radiotherapy combined with immunotherapy in NSCLC (*n* = 7). aPD-L1, anti–PD-L1; D, day. **N,** IHC was used to stain relevant markers in the NSCLC patient cohort and assess their correlation. Data represent the mean ± SD. Differences were tested using one-way ANOVA test (**A** and **E–M**), Wilcoxon rank-sum test (**C**), and unpaired two-sided Student *t* test (**N**). The correlation was determined using Spearman correlation test (**B** and **C**). *, *P* < 0.05; **, *P* < 0.01; ***, *P* < 0.001; ****, *P* < 0.0001.

Interestingly, the subcutaneous tumor model in BALB/c nude mice demonstrated a less pronounced impact of PRMT3 overexpression on tumor growth compared with the model in C57BL/6 mice (Fig. 4A). This observation has led us to hypothesize that the host’s immune response, particularly T cells, may account for the divergent outcomes associated with PRMT3 levels. Data analysis from TCGA supports this hypothesis, showing that PRMT3 is inversely correlated with T-cell infiltration across various tumor types, including LUSC and LUAD (Fig. 4B). Specifically, within NSCLC data, a robust negative correlation was observed between PRMT3 expression and the presence of CD8^+^ T cells, with lower infiltration of these cells in tumors exhibiting higher PRMT3 levels (Fig. 4C). Multiple IF analysis of mouse tumor samples also showed that high expression of PRMT3 was associated with fewer infiltrating CD8^+^ T cells (Fig. 4D). Flow cytometry analysis of mouse tumor tissues indicated that PRMT3 overexpression hinders the infiltration and functionality of CD8^+^ T cells after radiotherapy. Importantly, pharmacologic inhibition of IDO1 with 1-MT rescued the CD8^+^ T cells from the suppressive effects induced by PRMT3 overexpression (Fig. 4E and F). Furthermore, PRMT3 was found to upregulate Kyn levels within tumors, an effect that was mitigated by 1-MT treatment (Fig. 4G).

Current literature indicates that Kyn metabolism can influence T-cell behavior via several mechanisms. With this in mind, we conducted experiments to determine whether the impact of PRMT3 on CD8^+^ T cells operates through the IDO1-mediated Kyn metabolic pathway. We isolated CD8^+^ T cells from mouse spleens and cocultured them with LLC cells (Fig. 4H). Our results showed that PRMT3 overexpression in LLC cells increased Kyn levels in the culture medium, which corresponded with a reduction in the proportion of granzyme B–positive cells. Importantly, knocking down IDO1 can improve these effects, suggesting that low expression of IDO1 can reverse the suppression of CD8^+^ T-cell function by PRMT3 (Fig. 4I and J; Supplementary Fig. S8E). These findings indicate that PRMT3 may modulate the TME by enhancing IDO1 activity and Kyn production, which in turn affects CD8^+^ T-cell function and survival.

The activation of AhR has been identified as an essential component in the influence of Kyn on CD8^+^ T cells (31, 32). Our experiments revealed a notable upregulation of AhR activity in T cells exposed to PRMT3-overexpressing LLC cells, as demonstrated by the elevated mRNA levels of the quintessential AhR target genes, CYP1A1 and CYP1B1 (Fig. 4K). AhR, once activated, functions as a transcription factor within CD8^+^ T cells, capable of binding to the PD-1 promoter and augmenting PD-1 expression (33). We observed that PRMT3 overexpression in LLC cells led to increased PD-1 expression in cocultured T cells, an effect that was abrogated by knocking down IDO1 (Fig. 4L).

Inhibiting the intrinsic PRMT3 in tumor cells enhances their sensitivity to radiotherapy by suppressing Kyn metabolism. The release of Kyn into the TME subsequently hinders the infiltration and function of CD8^+^ T cells by activating the AhR pathway. Pharmacologic inhibition of PRMT3 not only amplifies the impact of radiotherapy but also, when combined with immunotherapy, further augments the efficacy of radiotherapy (Fig. 4M; Supplementary Fig. S8C). In our NSCLC cohort, patients were categorized as high expression or low expression based on the genes’ median expression. Patients in the PRMT3 (high) IDO1 (high) group exhibited reduced infiltration of CD8^+^ T cells than that in the PRMT3 (low) IDO1 (low) group (Fig. 4N; Supplementary Fig. S8D). These findings underscore the therapeutic potential of PRMT3 as a strategic target for radiosensitization, especially when combined with immunotherapy.

### PRMT3 catalyzes the methylation of TFAP2A to enhance the transcription of IDO1

**Figure 5.**
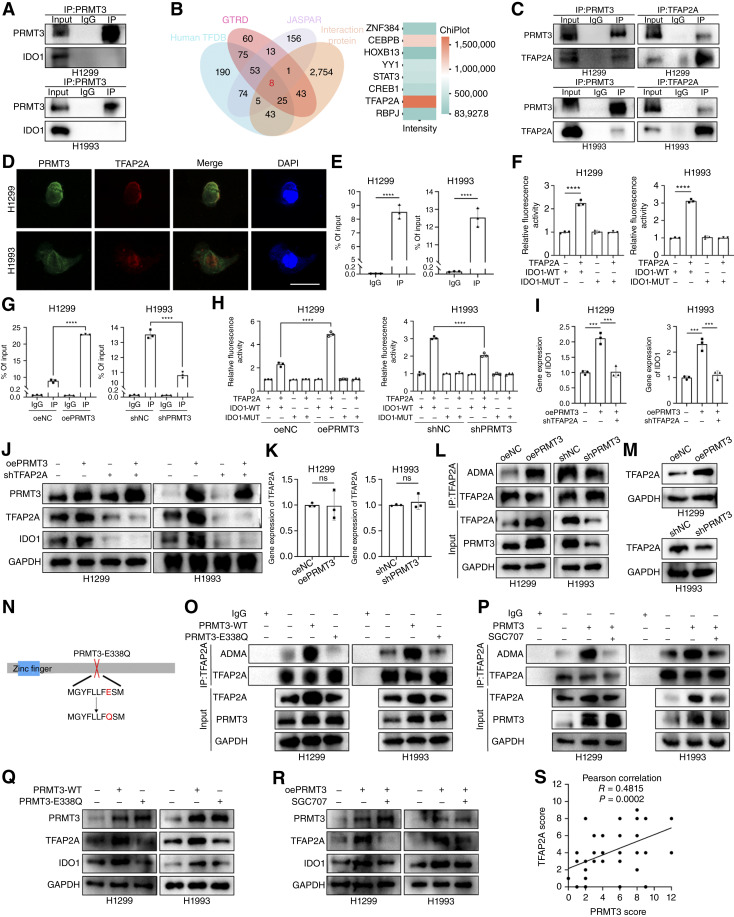
PRMT3 regulates IDO1 transcription through TFAP2A. **A,** Co-IP experiments showed that PRMT3 does not directly interact with IDO1. **B,** Predicted transcription factors of IDO1 intersected with PRMT3-interacting proteins. **C **and **D,** PRMT3 interacts with TFAP2A (**C**), and they colocalize (**D**). Scale bar, 20 μm. **E,** ChIP assays validated the binding of TFAP2A to the IDO1 promoter (*n* = 3). **F,** Luciferase assays indicated that TFAP2A is a transcription factor for IDO1 (*n* = 3). **G,** PRMT3 overexpression promoted the binding of TFAP2A to the IDO1 promoter (*n* = 3). **H** and **I,** PRMT3 knockdown inhibited TFAP2A binding to the IDO1 promoter, whereas TFAP2A knockdown rescued the PRMT3-mediated upregulation of IDO1 (*n* = 3). **J,** Overexpression of PRMT3 did not increase IDO1 expression in the absence of TFAP2A. **K,** PRMT3 did not affect TFAP2A mRNA levels (*n* = 3). **L **and **M,** Overexpression of PRMT3 promoted ADMA generation in TFAP2A (**L**), whereas PRMT3 influenced TFAP2A protein levels (**M**). **N,** Construction of PRMT3 enzyme inactivation mutant. **O,** Overexpression of PRMT3 with enzyme inactivation did not increase ADMA production in TFAP2A. **P,** Observation of the effect of treating cells with PRMT3 inhibitor on ADMA production in TFAP2A. **Q,** Western blot is used to detect the regulatory effect of enzyme inactivation mutants on TFAP2A protein levels. **R,** PRMT3 inhibitors can rescue the upregulation of TFAP2A protein levels caused by overexpression of PRMT3. **S,** There was a positive correlation between PRMT3 and TFAP2A expression in the NSCLC cohort. Data represent the mean ± SD. Differences were tested using unpaired two-sided Student *t* test (**E** and **K**) and one-way ANOVA test (**F**–**I**). **S,** The correlation was determined using Pearson correlation test. *, *P* < 0.05; **, *P* < 0.01; ***, *P* < 0.001; ****, *P* < 0.0001.

To explore the regulation of IDO1 by PRMT3, we performed MS analysis and IP. Our findings revealed that PRMT3 and IDO1 do not directly interact at the protein level (Fig. 5A). Given that PRMT3 influences IDO1 mRNA expression, we delved into the potential upstream regulators of IDO1 transcription. Utilizing bioinformatics tools from Human TFDB, GTRD, and JASPAR, we predicted the transcription factors for IDO1 and intersected this list with the PRMT3-interacting proteins identified via MS. This approach yielded eight candidate genes, with the TFAP2A emerging as the most prominent among the PRMT3 interactors (Fig. 5B). The interaction between PRMT3 and TFAP2A was confirmed through co-IP and IF analyses (Fig. 5C and D). Additionally, we demonstrated the binding of TFAP2A to the IDO1 promoter using dual-luciferase reporter assays and ChIP (Fig. 5E and F). We found that PRMT3 overexpression enhances the binding of TFAP2A to the IDO1 promoter, an effect reversed by PRMT3 knockdown (Fig. 5G and H). Moreover, TFAP2A knockdown negated the upregulatory effect of PRMT3 overexpression on IDO1 mRNA (Fig. 5I). The protein level of IDO1 is diminished in cells in which TFAP2A is knocked down, and even with PRMT3 overexpression, there is no increase in IDO1 levels (Fig. 5J). The upregulation of IDO1 by PRMT3 depends on TFAP2A.

Notably, PRMT3 levels did not alter TFAP2A mRNA levels (Fig. 5K), suggesting a posttranscriptional regulatory mechanism. Our data indicated that PRMT3 overexpression facilitates the ADMA formation in TFAP2A. Changes in PRMT3 levels were associated with corresponding fluctuations in ADMA signals on TFAP2A, implicating the catalytic activity of PRMT3 (Fig. 5L). PRMT3 knockdown reduced TFAP2A protein expression (Fig. 5M). To probe the role of PRMT3 enzymatic activity on TFAP2A, we used both wild-type PRMT3 and a catalytically inactive mutant, PRMT3-E338Q (Fig. 5N; Supplementary Fig. S8G). The inactive mutant PRMT3 cannot catalyze the formation of ADMA in TFAP2A, and it also does not facilitate its protein upregulation (Fig. 5O and Q). Consistently, pharmacologic inhibition of PRMT3 enzymatic activity recapitulated these findings (Fig. 5P and R). In our NSCLC cohort, the expression of TFAP2A was significantly positively correlated with PRMT3 (Fig. 5S; Supplementary Fig. S8F).

### PRMT3 methylated TFAP2A at R363 and affected the half-life, nuclear localization, and dimer formation of TFAP2A

**Figure 6.**
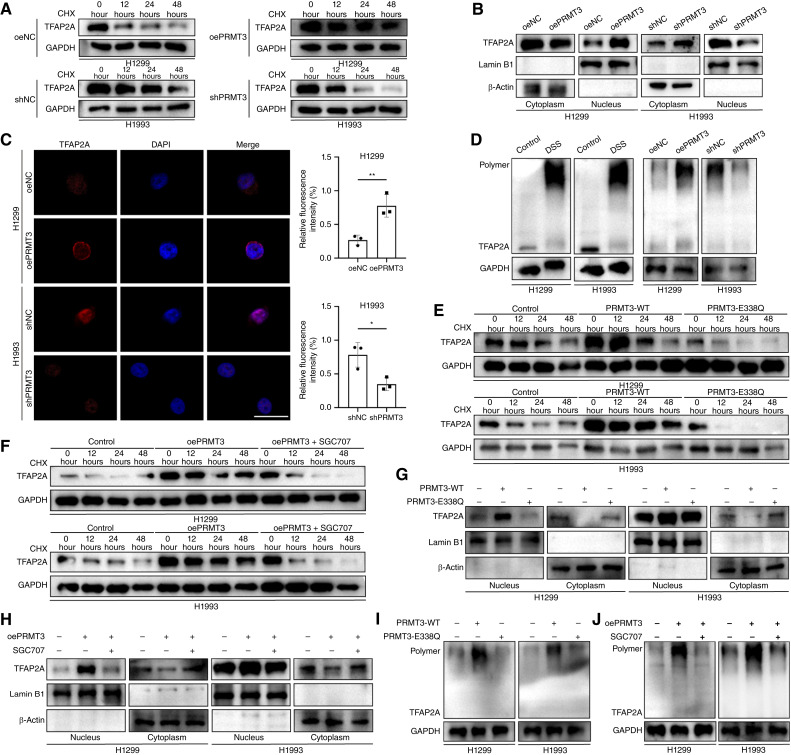
PRMT3-mediated methylation extends TFAP2A protein stability. **A** and **B,** PRMT3 overexpression prolonged TFAP2A half-life and promoted nuclear uptake. **C,** IF revealed that PRMT3 regulates TFAP2A nuclear localization (*n* = 3). Scale bar, 20 μm. **D,** Disuccinimidyl suberate (DSS) assays showed that PRMT3 knockdown inhibited TFAP2A dimerization. **E–J, **PRMT3 enzyme-inactivated mutants did not affect TFAP2A half-life (**E**), nuclear uptake (**G**), and dimer formation (**I**). The PRMT3 inhibitor SGC707 rescued the effects of PRMT3 overexpression on TFAP2A half-life (**F**), nuclear localization (**H**), and dimer formation (**J**). Data represent the mean ± SD. Differences were tested using unpaired two-sided Student *t* test (**C**). *, *P* < 0.05; **, *P* < 0.01. CHX, cycloheximide.

Given that PRMT3 enhances the expression of the TFAP2A protein in cells, we investigated its impact on the protein’s half-life. Western blot analysis revealed that the overexpression of PRMT3 extends the half-life of TFAP2A (Fig. 6A). Furthermore, the nuclear localization of TFAP2A was found to be diminished upon PRMT3 knockdown (Fig. 6B), with an inverse trend observed in cells overexpressing PRMT3, in which more TFAP2A protein was directed to the nucleus (Fig. 6C). TFAP2A operates within cells through the formation of homodimers or heterodimers (34). Utilizing a disuccinimidyl suberate cell cross-linking assay, we confirmed that PRMT3 fosters the assembly of TFAP2A dimers in cells (Fig. 6D). However, the overexpression of a catalytically inactive PRMT3 mutant, PRMT3-E338Q, failed to extend the half-life, augment nuclear entry, or facilitate dimer formation (Fig. 6E, G, and I). In line with these discoveries, the pharmacologic inhibition of PRMT3 enzyme activity similarly suppresses the half-life, nuclear localization, and dimerization of TFAP2A (Fig. 6F, H, and J).

**Figure 7.**
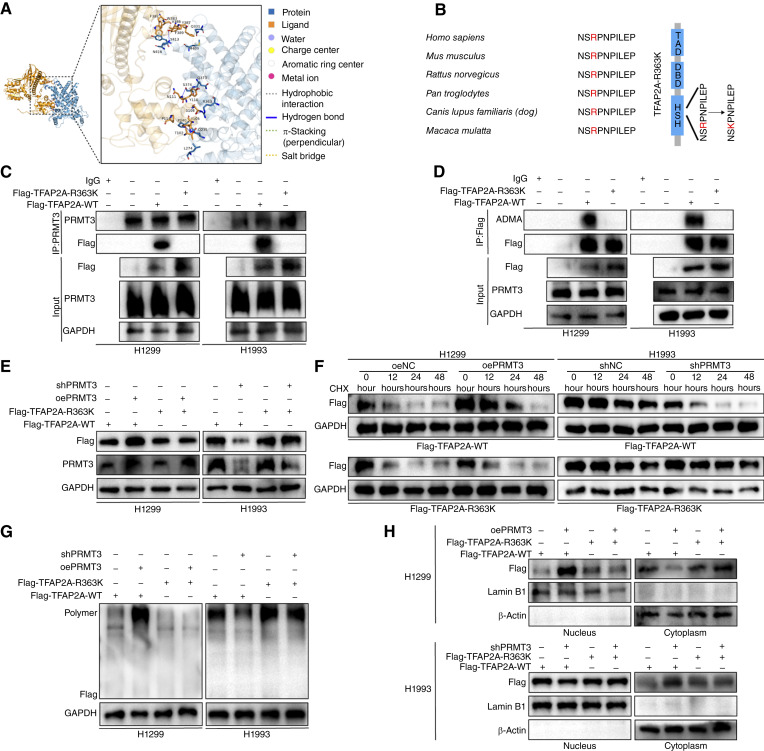
PRMT3 methylates TFAP2A at R363. **A,** Molecular docking simulations were conducted to predict the potential methylation sites of TFAP2A. **B,** In mammals, the amino acid sequence surrounding the R363 site of TFAP2A is highly conserved. A mutant with the substitution R363K was constructed. DBD, DNA-binding domain; TAD, transactivation domain. **C,** PRMT3 was unable to directly interact with the mutant proteins.** D–H, **The mutant proteins were incapable of generating ADMA (**D**), and PRMT3 could not regulate their protein levels (**E**), half-life (**F**), dimerization (**G**), or nuclear localization (**H**). CHX, cycloheximide.

The TFAP2A protein is structured with distinct functional domains, including a transactivation domain, a DNA-binding domain, and two helix-span-helix (HSH) domains. Notably, these HSH domains are linked through hydrophobic interactions to form a stable dimer (35). To elucidate the interaction mechanism between PRMT3 and TFAP2A, we used protein–protein molecular docking simulation analysis. This computational approach predicted an interaction interface and identified a critical arginine residue, R363, within the HSH domain of TFAP2A as a potential binding site for PRMT3 (Fig. 7A). The peptide segment encompassing R363 was found to be highly conserved across various mammalian species. To test the functional significance of this interaction, we engineered a mutation wherein arginine at position 363 of TFAP2A was substituted with lysine, creating the R363K mutant (Fig. 7B; Supplementary Fig. S8H and S8I). Our findings demonstrate that PRMT3 was unable to interact with and methylate the R363K mutant of TFAP2A (Fig. 7C and D). Consequently, overexpression or knockdown of PRMT3 failed to influence the protein expression, half-life, nuclear localization, and dimer formation of the K363-TFAP2A mutant (Fig. 7E–H).

### Targeting the PRMT3–IDO1 axis enhances the efficacy of radiotherapy in NSCLC

**Figure 8.**
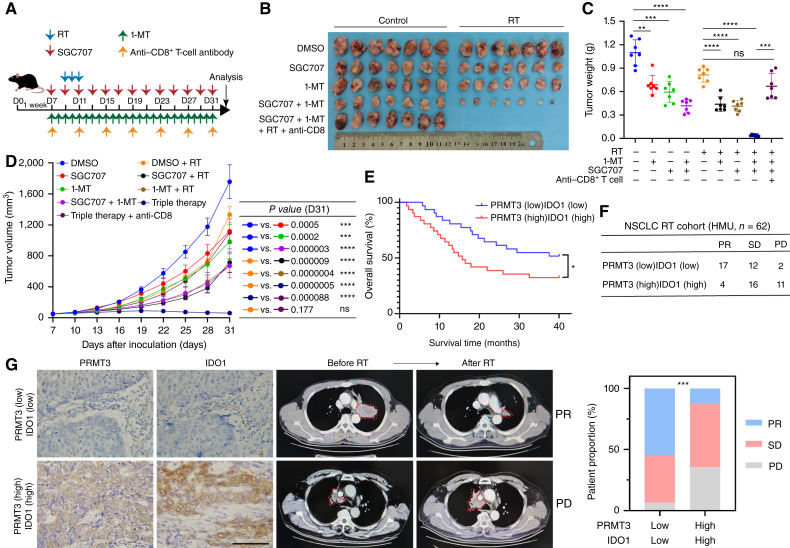
Targeting the PRMT3–IDO1 axis sensitizes radiotherapy in NSCLC. **A–D,** Mouse xenograft models demonstrated that the combination of PRMT3 and IDO1 inhibitors enhanced the sensitization of radiotherapy (RT). Tumor growth, weight, and survival were compared among different groups (*n* = 7). D, day. **E,** Patient survival data were stratified by different expression levels of PRMT3 and IDO1. **F** and **G,** The therapeutic efficacy was assessed in an NSCLC patient cohort. HMU, Harbin Medical University; SD, stable disease. Data represent the mean ± SD. Differences were tested using one-way ANOVA test (**C** and **D**), log-rank test (**E**), and Pearson χ^2^ test (**F**). ns, not significant; **, *P* < 0.01; ***, *P* < 0.001; ****, *P* < 0.0001.

Compared with radiotherapy alone, the combined use of SGC707 or 1-MT significantly suppressed tumor growth. Among these, the combination of SGC707 and 1-MT demonstrated the most pronounced radiosensitizing effect. More importantly, the benefit of SGC707/1-MT/radiotherapy triple-therapy was partially eliminated after anti-CD8 treatment (Fig. 8A–D). In our NSCLC cohort, the PRMT3 (low) IDO1 (low) group showed superior overall survival compared with the PRMT3 (high) IDO1 (high) group (Fig. 8E; Supplementary Table S10). The PRMT3 (low) IDO1 (low) group showed a higher proportion of sensitivity to radiotherapy (Fig. 8F and G). In conclusion, our findings suggest that PRMT3–IDO1 levels can predict the radiotherapeutic response in patients with NSCLC. Targeting the PRMT3–IDO1–Kyn metabolism axis enhances the radiotherapeutic effect in NSCLC.

## Discussion

Radiation resistance in NSCLC poses a significant clinical challenge, impairing the effectiveness of radiotherapy. This resistance can result from various factors, including the intrinsic radiation resistance of cancer cells, the TME, hypoxia, and the capacity of cancer cells to repair radiation-induced damage (36–38). The effectiveness of radiotherapy differs among patients, with their specific gene expression profiles potentially determining their response to treatment. Exploring biomarker targets for radiotherapy resistance could be an effective approach to tackle the issue of radiotherapy resistance. It has been reported that arginine can influence tumor therapeutic resistance (26, 39–42). However, despite this, the role of PRMT3 in modulating radiosensitivity remains to be explored. Our study reveals that in NSCLC cells, upregulated PRMT3 promotes IDO1 expression by methylating TFAP2A, thereby activating the tumor’s intrinsic Kyn metabolism and mediating the development of radiation resistance.

Metabolic reprogramming is a hallmark of cancer. Moreover, alterations in tumor metabolism can modify disease characteristics and foster treatment resistance (43, 44). Metabolite-focused targeted therapy has proven effective in enhancing the efficacy of radiotherapy (45). Consequently, there is a need to systematically delineate the gene metabolic landscape during cancer therapy. Current research indicates that lower Kyn related markers following radiotherapy are associated with improved survival in patients with early-stage NSCLC (46). Metabolites within the Kyn metabolic pathway are attributed with radioprotective properties (47). Activation of AhR by Kyn influences cell death through modulation of ROS production and stimulates the expression of tumor stemness genes (48–50). Additionally, the Kyn metabolic pathway, serving as a significant source of NAD+, can modulate DNA damage response (51). Our study underscores that high intrinsic tumor PRMT3 expression can elevate Kyn metabolism, facilitating the development of radiotherapy resistance.

Immune cells and tumor cells share many metabolic pathways, and tumor metabolic reprogramming not only affects the tumor cells themselves but also influences tumor immunity by regulating metabolites within the TME (52). Previous studies have demonstrated that inhibiting the Kyn metabolic pathway not only reduces tumor metastasis and resistance to apoptosis but also enhances CD8^+^ T-cell function (33, 53, 54). Kyn, as the primary endogenous ligand for AHR activation, and the AhR-dependent negative feedback can suppress STING signaling and IFNI production (32). The subpopulation of cancer-associated fibroblasts positive for podocin utilizes the Kyn metabolic pathway to inhibit antibody-dependent cell-mediated cytotoxicity (55). Tobacco smoke is believed to induce Kyn metabolism, thereby promoting immune suppression and contributing to lung cancer development (56). Blocking the interaction between Kyn and CD4^+^ T cells can inhibit regulatory T-cell differentiation, enhance CD8^+^ T-cell function, and significantly boost in vivo anti–PD-1 therapeutic efficacy (57). Our findings indicate that targeting PRMT3 in tumor cells and inhibiting Kyn metabolism not only suppresses tumor growth but also significantly mitigates the development of an immunosuppressive microenvironment by activating AhR.

Among the enzymes in the Kyn metabolic pathway, we identified a significant role for IDO1 in PRMT3-mediated radiotherapy resistance. PRMT3-induced arginine methylation facilitates the nuclear translocation and dimerization of the IDO1 transcription factor TFAP2A, extending its half-life. Consequently, in tumor cells with upregulated PRMT3, IDO1 transcription is enhanced, thereby activating Kyn metabolism. The elevated levels of Kyn in the TME activate the AhR in CD8^+^ T cells, which subsequently suppresses their infiltration and cytotoxic function, while also leading to the upregulation of PD-1 expression. The combined pharmacologic inhibition of PRMT3 and IDO1 maximized the radiosensitizing effect of radiotherapy. Additionally, PRMT3’s regulatory influence on the tumor-immune microenvironment suggests its potential in radiotherapy combined with immunotherapy.

Emerging research indicates that varying doses and fractionation schemes of radiotherapy may be associated with distinct oncocellular metabolism and immune microenvironments (58). Researches have revealed that stereotactic body radiotherapy is superior to conventional radiotherapy due to its more potent immune-activating effects in various solid tumors. Wang and colleagues (59) further demonstrated that in patients with early-stage NSCLC, stereotactic body radiotherapy exerts a less immunosuppressive effect compared with three-dimensional conformal radiotherapy, as evidenced by lower IDO expression. Therefore, elucidating the optimal dosing and fractionation regimen for radiotherapy combined with PRMT3 and IDO1 inhibitors will be a pivotal inquiry that we intend to investigate in our forthcoming research endeavors.

## Supplementary Material

Supplementary Figure S1 PRMT3 as a key arginine methyltransferase in NSCLC.

Supplementary Figure S2 PRMT3 regulates NSCLC cell proliferation and migration post-radiotherapy.

Supplementary Figure S3 In vivo and in vitro validation of PRMT3's role in promoting radiotherapy resistance in NSCLC.

Supplementary Figure S4 PRMT3 regulates NSCLC radiotherapy resistance through Kyn metabolism.

Supplementary Figure S5 The effect of PRMT3 on NSCLC depends on IDO1.

Supplementary Figure S6 PRMT3-IDO1 axis affects NSCLC cell proliferation after radiotherapy.

Supplementary Figure S7 Validation of the PRMT3-IDO1 axis through in vivo and in vitro experiments.

Supplementary Figure S8 The PRMT3-IDO1 axis modulates the immune microenvironment in NSCLC.

Supplementary Table S1 The details of the detection antibodies involved in the study.

Supplementary Table S2 The lists of primers used for qRT-PCR or CHIP-qPCR.

Supplementary Table S3 The sequences of shRNAs.

Supplementary Table S4 The antibody panels for sorting T cells by flow cytometry.

Supplementary Table S5 The sequence of wild type and mutant type of IDO1 in luciferase reporter assay.

Supplementary Table S6 The antibodies utilized in multiplex immunofluorescence assays.

Supplementary Table S7 Clinical characteristics of NSCLC patients for qPCR of PRMT3.

Supplementary Table S8 Expression differences of PRMT family genes in samples from the GSE25814 dataset before and after radiotherapy.

Supplementary Table S9 Clinical characteristics of NSCLC patients for the IDO-PRMT3 correlation.

Supplementary Table S10 Clinical characteristics of NSCLC patients for survival data.

## Data Availability

The previously published transcriptomic data analyzed in this study were obtained from Gene Expression Omnibus at GSE25814. The genetic mutation and RNA-seq datasets were sourced from the TCGA database (TCGA-LUAD and TCGA-LUSC), accessible through the Genomic Data Commons (https://portal.gdc.cancer.gov/). The RNA-seq data generated in this study are available in the National Center for Bioinformation GSA-Human database (https://ngdc.cncb.ac.cn/gsa-human/) at HRA013513. All requests for the data will be granted by the Data Access Committee. All other raw data generated in this study are available upon request to the corresponding author.
