# Supplementary material for: PRMT3 Drives IDO1-Dependent Radioresistance and Immunosuppression by Promoting Kynurenine Metabolism in Non–Small Cell Lung Cancer
Source: Cancer Res. 2025 Oct 23;86(2):421–37. doi: 10.1158/0008-5472.CAN-24-4162 (PMC12809119; doi:10.1158/0008-5472.CAN-24-4162)
Supplement: Supplementary Figure S1 — PRMT3 as a key arginine methyltransferase in NSCLC. [file can-24-4162_supplementary_figure_s1_suppsf1.pdf]

Supplementary Figure S1

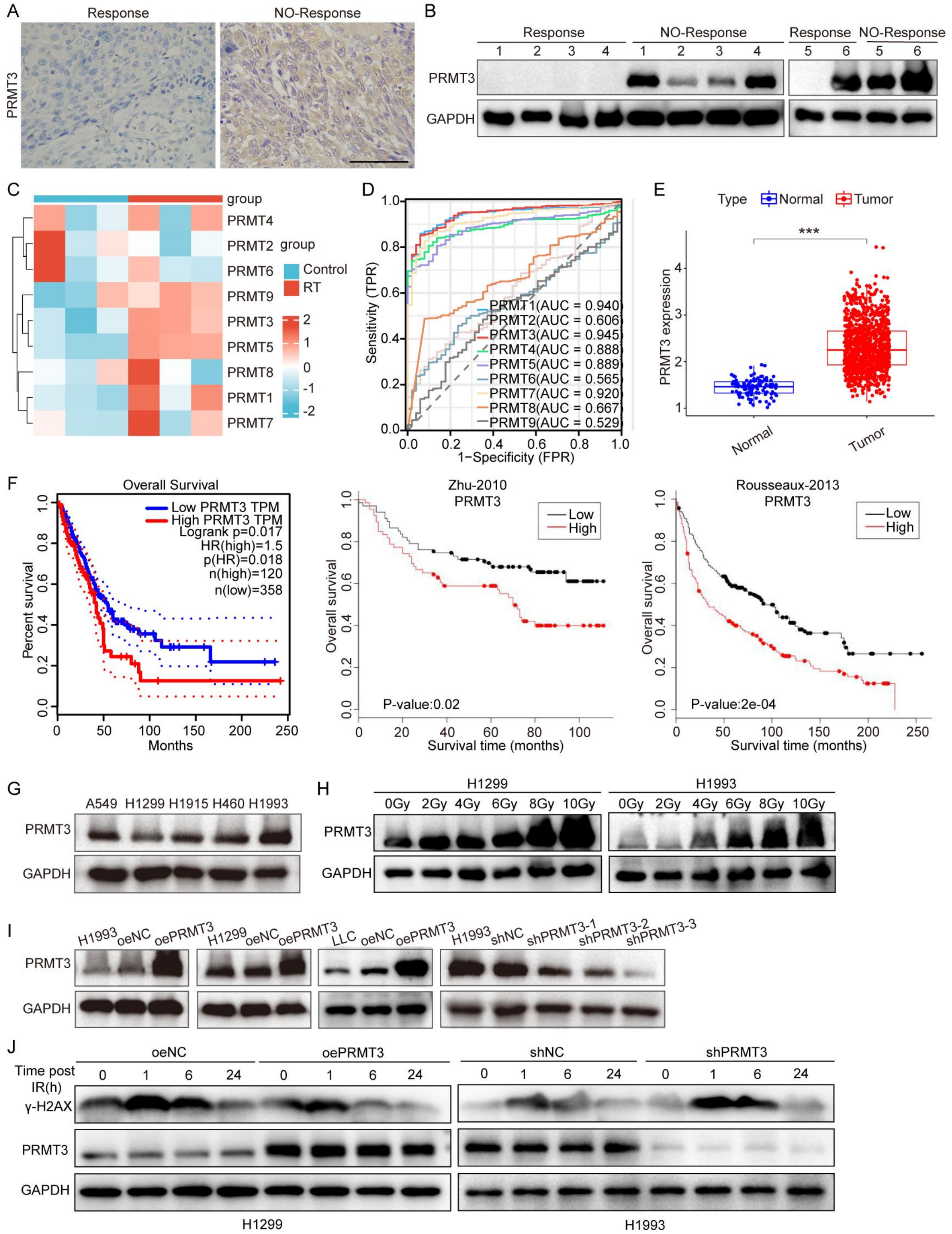

Supplementary Figure S1 PRMT3 as a key arginine methyltransferase in NSCLC.

(A) IHC staining for PRMT3 in NSCLC patients with varying responses to radiotherapy. Scale bar: 100 μm. (B) Western blot analysis of PRMT3 expression in

12 patients from both the responsive and non-responsive groups. (C) A heatmap displaying PRMT family gene expression in radiotherapy-treated and untreated cells from the GSE25814 dataset. NSCLC cells were harvested 96 hours after exposure to 5 Gy ionizing radiation. (D) Receiver operating characteristic (ROC) curve analysis using TCGA LUAD and LUSC data. (E) PRMT3 expression in NSCLC versus normal tissues from the TCGA database. (F) NSCLC patients with elevated PRMT3 expression had worse survival outcomes. (G) Western blotting was employed to assess PRMT3 expression across established NSCLC cell lines. (H) PRMT3 expression increased in a dose-dependent manner following radiotherapy. (I) Cell lines with PRMT3 overexpression and knockdown were established. (J) Western blotting detected dynamic changes in γ-H2AX expression at different time points (0, 1, 6, and 24 hours) after irradiation (6 Gy). Data represent the mean ± SD. Differences were tested using 1-way ANOVA test (C-D), Log-rank test (F).
