# Supplementary material for: PRMT3 Drives IDO1-Dependent Radioresistance and Immunosuppression by Promoting Kynurenine Metabolism in Non–Small Cell Lung Cancer
Source: Cancer Res. 2025 Oct 23;86(2):421–37. doi: 10.1158/0008-5472.CAN-24-4162 (PMC12809119; doi:10.1158/0008-5472.CAN-24-4162)
Supplement: Supplementary Figure S2 — PRMT3 regulates NSCLC cell proliferation and migration post-radiotherapy. [file can-24-4162_supplementary_figure_s2_suppsf2.pdf]

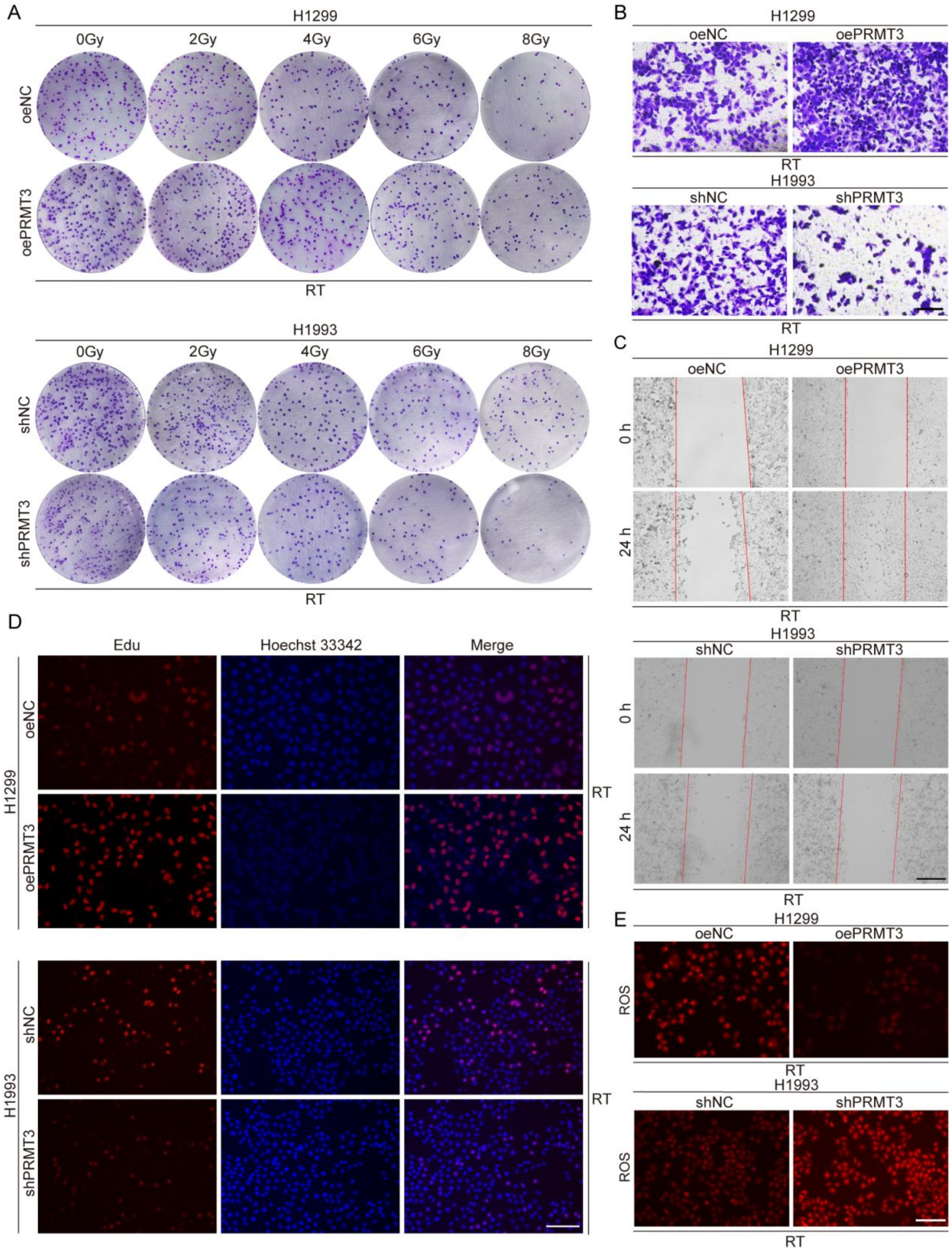

Supplementary Figure S2 PRMT3 regulates NSCLC cell proliferation and migration post-radiotherapy.

(A) Clonogenic survival assays (0-8 Gy), (B) transwell migration assays (4 Gy; scale bar: 100 μm), (C) wound healing assays (4 Gy; scale bar: 200 μm) were performed on

radiotherapy-treated NSCLC cells. (D) EdU incorporation (4 Gy; scale bar: 50 μm) and (E) ROS (6 Gy; scale bar: 50 μm) production were measured following radiotherapy.
