# Supplementary material for: PRMT3 Drives IDO1-Dependent Radioresistance and Immunosuppression by Promoting Kynurenine Metabolism in Non–Small Cell Lung Cancer
Source: Cancer Res. 2025 Oct 23;86(2):421–37. doi: 10.1158/0008-5472.CAN-24-4162 (PMC12809119; doi:10.1158/0008-5472.CAN-24-4162)
Supplement: Supplementary Figure S3 — In vivo and in vitro validation of PRMT3's role in promoting radiotherapy resistance in NSCLC. [file can-24-4162_supplementary_figure_s3_suppsf3.pdf]

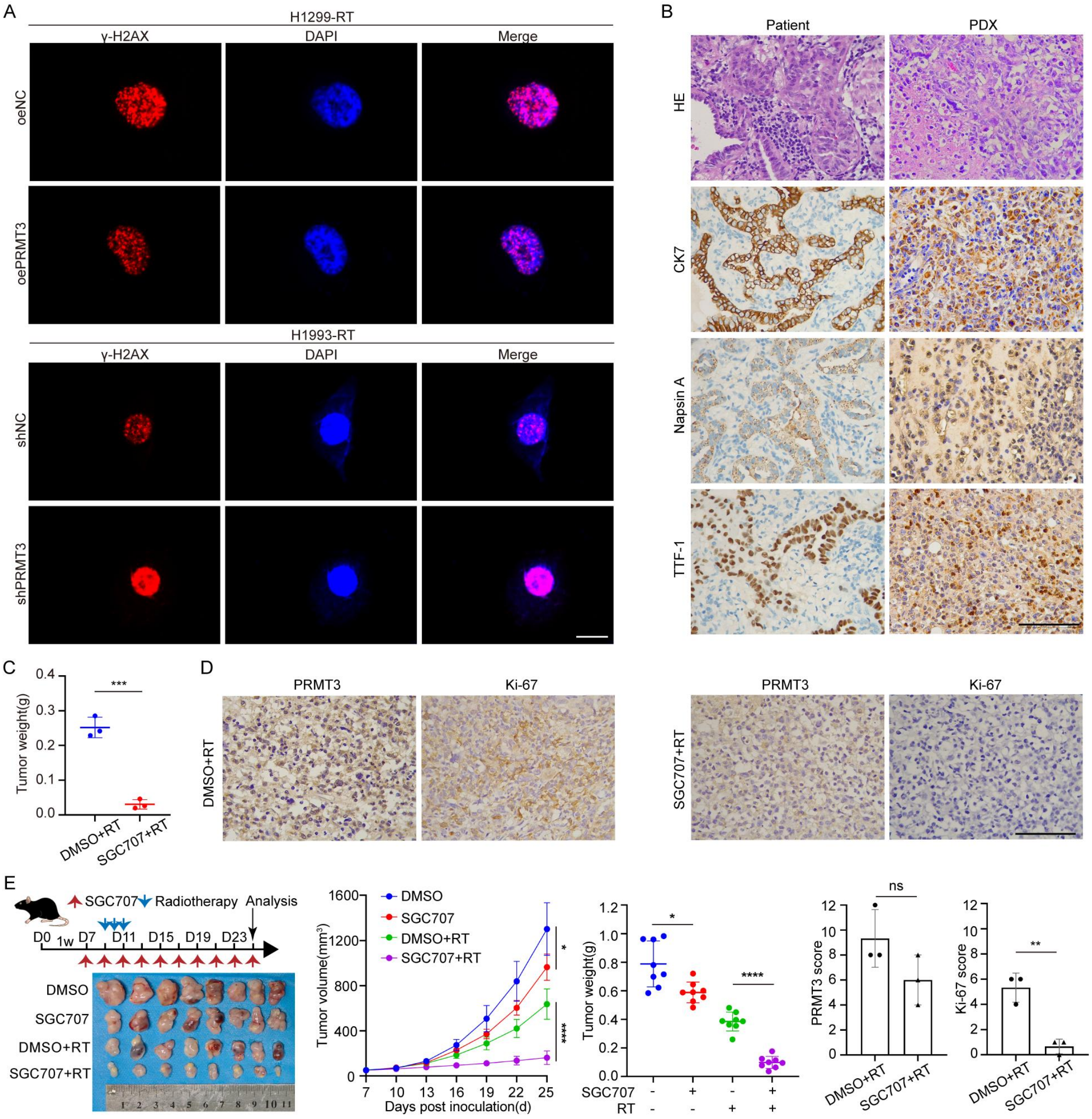


**Supplementary Figure S3 In vivo and in vitro validation of PRMT3's role in promoting radiotherapy resistance in NSCLC.**

(A) Overexpression of PRMT3 reduced γ-H2AX production following radiotherapy (6 Gy, Scale bar: 20 μm). (B) H&E and IHC staining of target proteins in patient samples and corresponding mouse tumors from PDX. Scale bar: 100 μm. (C) Tumor weight of PDX mice (n=3). (D) IHC staining of target proteins in mouse tumor samples. Scale bar: 100 μm. (E) Representative images, statistical analyses of the tumor volume and tumor weight of xenograft tumors in C57BL/6 mice (n=8). Data

represent the mean ± SD. *$P < 0.05$, **$P < 0.01$, ***$P < 0.001$ and ****$P < 0.0001$. Differences were tested using unpaired 2-sided Student's t test (C-D), 1-way ANOVA test (E).
