# Supplementary material for: PRMT3 Drives IDO1-Dependent Radioresistance and Immunosuppression by Promoting Kynurenine Metabolism in Non–Small Cell Lung Cancer
Source: Cancer Res. 2025 Oct 23;86(2):421–37. doi: 10.1158/0008-5472.CAN-24-4162 (PMC12809119; doi:10.1158/0008-5472.CAN-24-4162)
Supplement: Supplementary Figure S4 — PRMT3 regulates NSCLC radiotherapy resistance through Kyn metabolism. [file can-24-4162_supplementary_figure_s4_suppsf4.pdf]

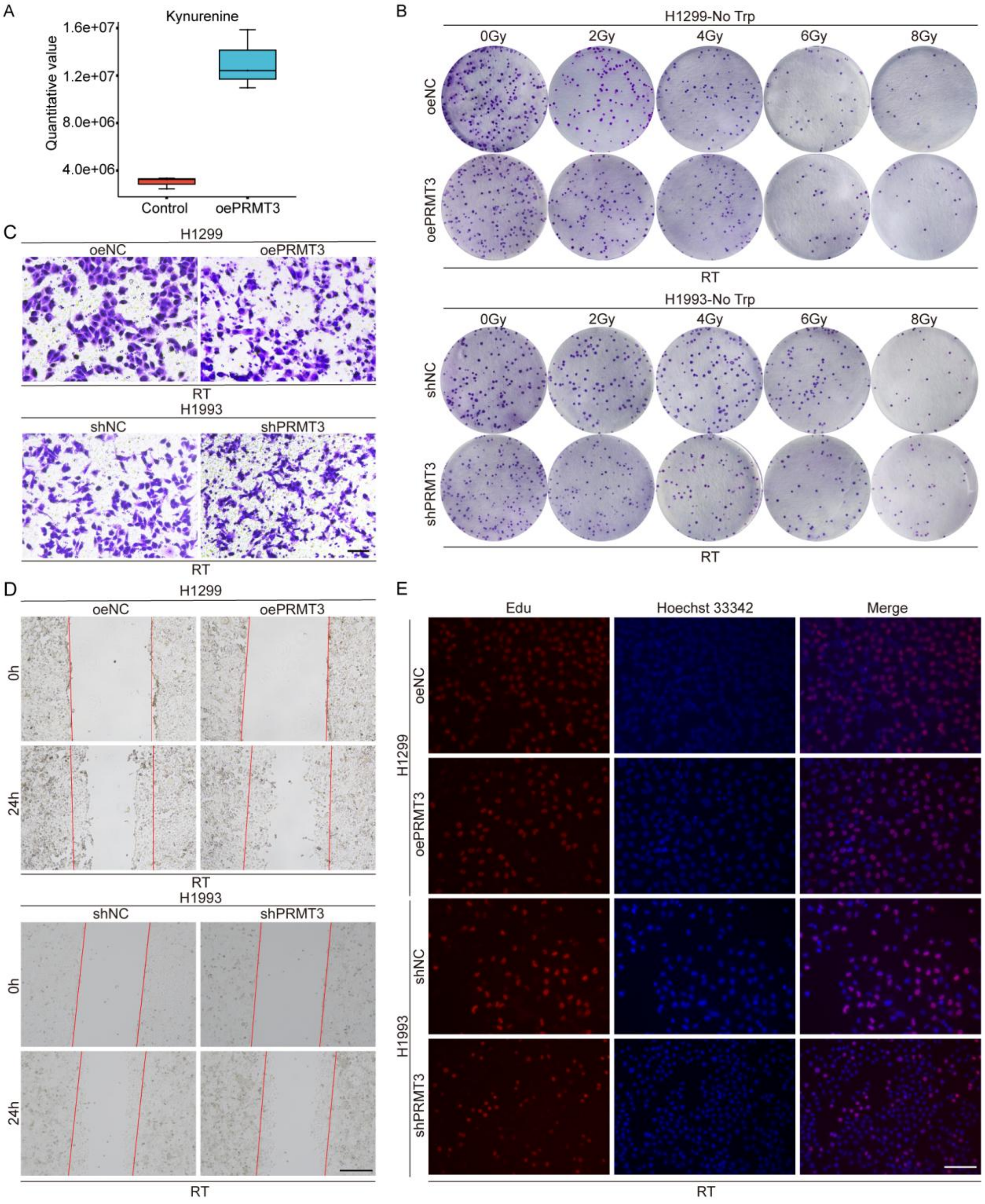

Supplementary Figure S4 PRMT3 regulates NSCLC radiotherapy resistance through Kyn metabolism.

(A) Kyn levels were elevated in PRMT3-overexpressing cells. In the absence of tryptophan, PRMT3 could not regulate NSCLC cell (B) clonogenic survival (0-8 Gy), (C) transwell (4 Gy; scale bar: 100 μm), (D) wound healing (4 Gy; scale bar: 200 μm) and form (E) EdU incorporation (4 Gy; scale bar: 50 μm) after radiotherapy.
