# Supplementary material for: PRMT3 Drives IDO1-Dependent Radioresistance and Immunosuppression by Promoting Kynurenine Metabolism in Non–Small Cell Lung Cancer
Source: Cancer Res. 2025 Oct 23;86(2):421–37. doi: 10.1158/0008-5472.CAN-24-4162 (PMC12809119; doi:10.1158/0008-5472.CAN-24-4162)
Supplement: Supplementary Figure S5 — The effect of PRMT3 on NSCLC depends on IDO1. [file can-24-4162_supplementary_figure_s5_suppsf5.pdf]

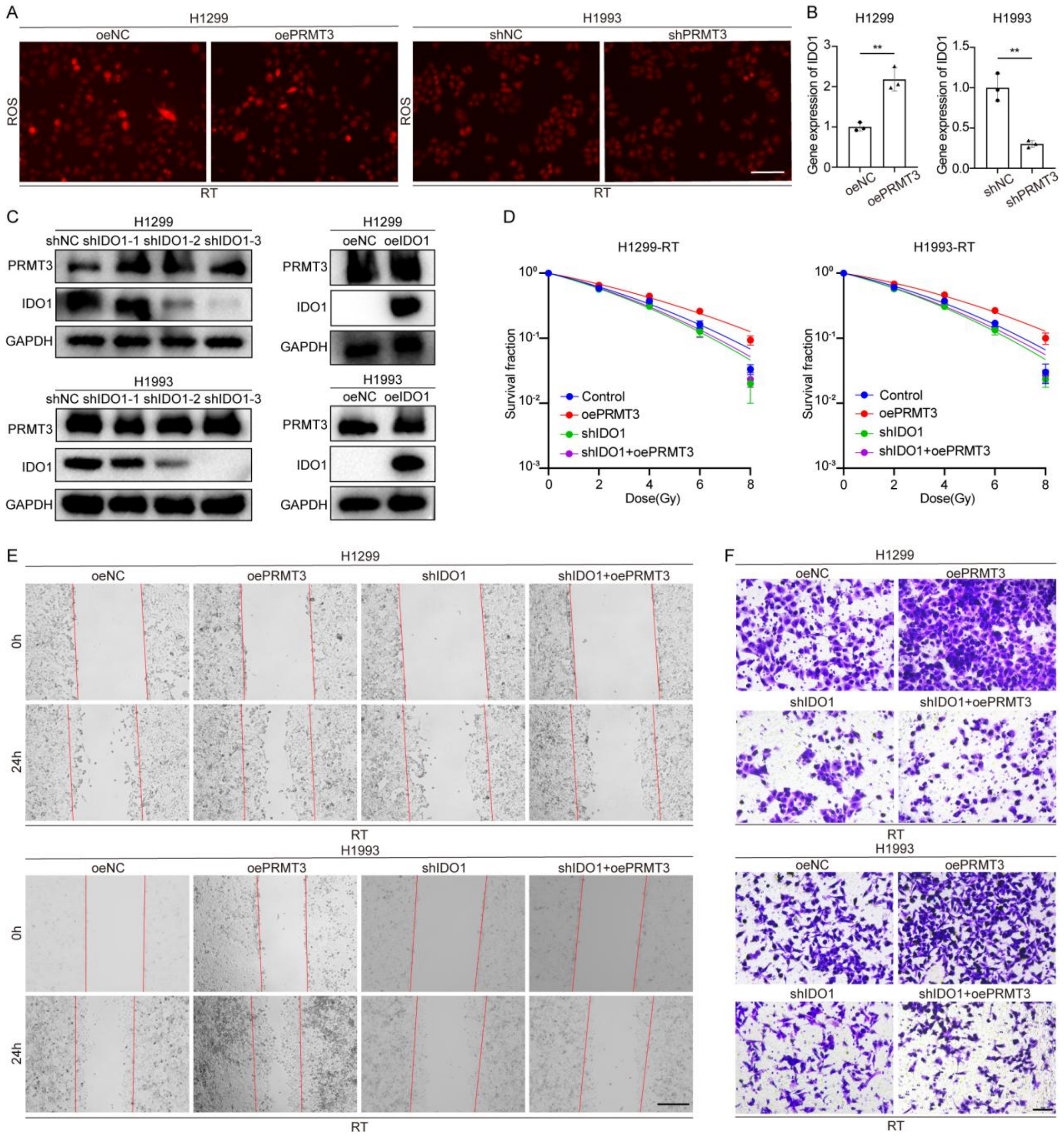

Supplementary Figure S5 The effect of PRMT3 on NSCLC depends on IDO1.

(A) In tryptophan-deficient culture conditions, PRMT3 expression did not impact ROS production following radiotherapy (6 Gy). Scale bar: 50 μm. (B) PCR validated PRMT3-induced upregulation of IDO1 at mRNA level (n=3). (C) IDO1 knockdown and overexpression cell lines were established. (D) IDO1 knockdown reversed the proliferative effect of PRMT3 overexpression, and (E-F) inhibited migration (4 Gy; scale bars: 100 μm for E and 200 μm for F) in NSCLC cells after radiotherapy. Data represent the mean ± SD. *$P < 0.05$, **$P < 0.01$, ***$P < 0.001$ and ****$P < 0.0001$.

Differences were tested using unpaired 2-sided Student's t test (B).
