# Supplementary material for: PRMT3 Drives IDO1-Dependent Radioresistance and Immunosuppression by Promoting Kynurenine Metabolism in Non–Small Cell Lung Cancer
Source: Cancer Res. 2025 Oct 23;86(2):421–37. doi: 10.1158/0008-5472.CAN-24-4162 (PMC12809119; doi:10.1158/0008-5472.CAN-24-4162)
Supplement: Supplementary Figure S6 — PRMT3-IDO1 axis affects NSCLC cell proliferation after radiotherapy. [file can-24-4162_supplementary_figure_s6_suppsf6.pdf]

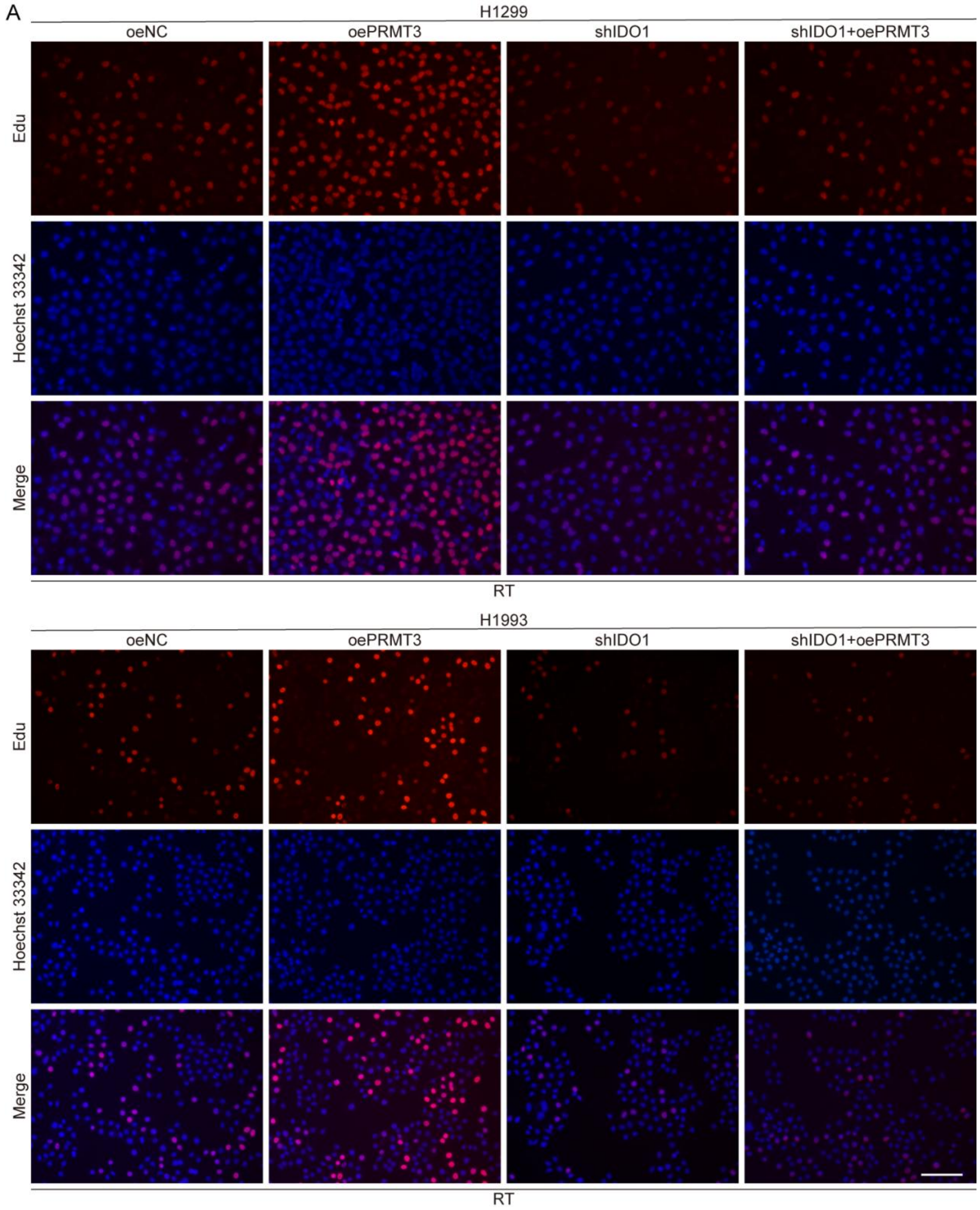


**Supplementary Figure S6 PRMT3-IDO1 axis affects NSCLC cell proliferation after radiotherapy.**

(A) EdU incorporation assays confirmed that PRMT3's effect on NSCLC depends on IDO1 (4 Gy). Scale bar: 50 μm.
