# Supplementary material for: PRMT3 Drives IDO1-Dependent Radioresistance and Immunosuppression by Promoting Kynurenine Metabolism in Non–Small Cell Lung Cancer
Source: Cancer Res. 2025 Oct 23;86(2):421–37. doi: 10.1158/0008-5472.CAN-24-4162 (PMC12809119; doi:10.1158/0008-5472.CAN-24-4162)
Supplement: Supplementary Figure S7 — Validation of the PRMT3-IDO1 axis through in vivo and in vitro experiments. [file can-24-4162_supplementary_figure_s7_suppsf7.pdf]

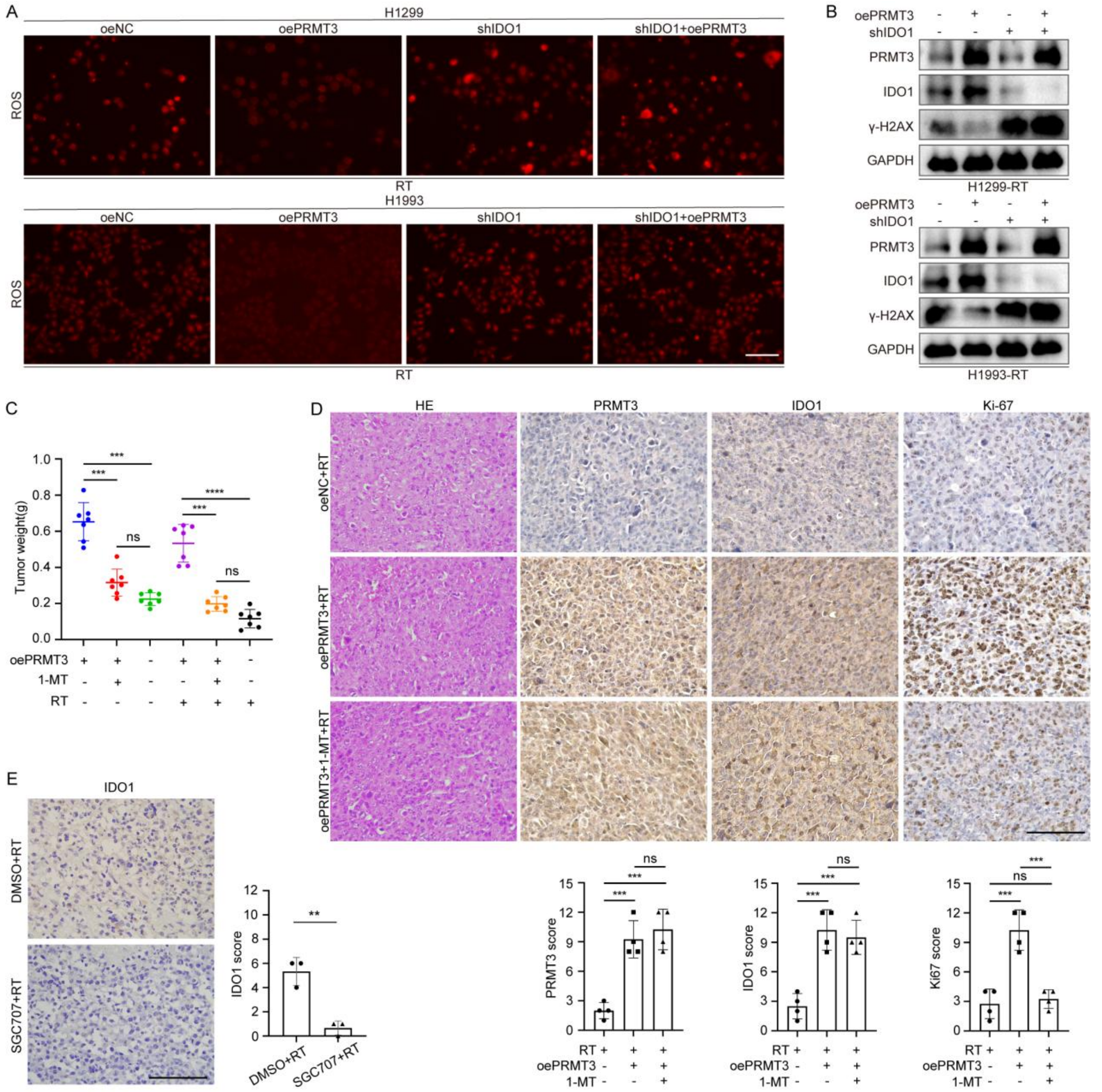

Supplementary Figure S7 Validation of the PRMT3-IDO1 axis through in vivo and in vitro experiments.

(A-B) In IDO1 knockdown conditions, PRMT3 overexpression failed to induce ROS production or γ-H2AX formation following radiotherapy (6 Gy). Scale bar: 50 μm. (C) IDO1 inhibitors reversed PRMT3-mediated tumor resistance to radiotherapy in mice. (D) H&E and IHC staining were performed on tumors from different mouse groups. Scale bars: 100 μm. (E) In the PDX model, tumors in the SGC707 treatment group exhibited lower IDO1 expression. Scale bar: 100 μm. Data represent the mean ± SD. **P* < 0.05, ***P* < 0.01, ****P* < 0.001 and *****P* < 0.0001. Differences were tested

using 1-way ANOVA test (C-D) and unpaired 2-sided Student's t test (E).
