# Supplementary material for: PRMT3 Drives IDO1-Dependent Radioresistance and Immunosuppression by Promoting Kynurenine Metabolism in Non–Small Cell Lung Cancer
Source: Cancer Res. 2025 Oct 23;86(2):421–37. doi: 10.1158/0008-5472.CAN-24-4162 (PMC12809119; doi:10.1158/0008-5472.CAN-24-4162)
Supplement: Supplementary Figure S8 — The PRMT3-IDO1 axis modulates the immune microenvironment in NSCLC. [file can-24-4162_supplementary_figure_s8_suppsf8.pdf]

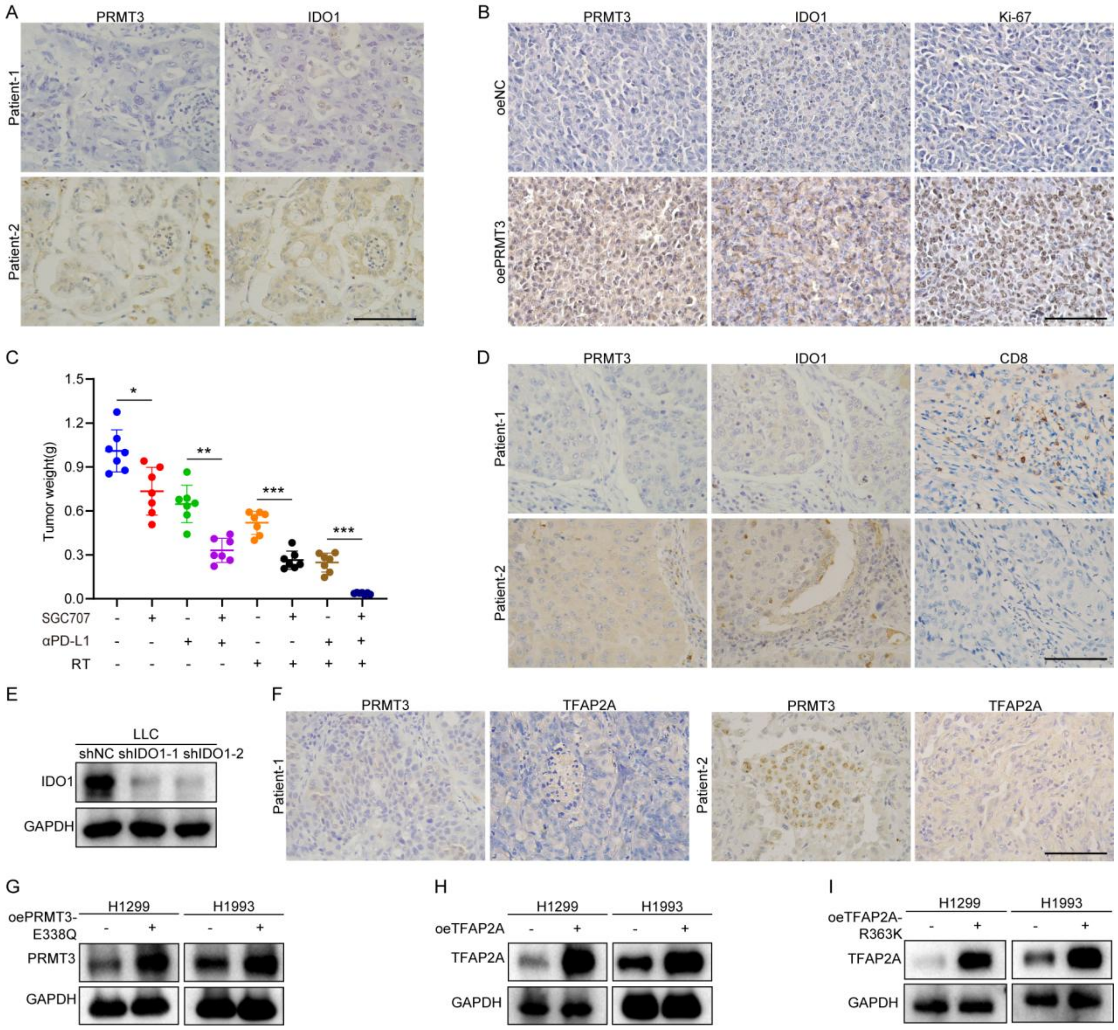

Supplementary Figure S8 The PRMT3-IDO1 axis modulates the immune microenvironment in NSCLC

(A) IHC staining was used to evaluate the expression of target proteins in NSCLC patients. Scale bar: 100 μm. (B) IHC assessed PRMT3 and IDO1 expression in tumor samples. Scale bar: 100 μm. (C) In vivo experiments verified the effect of PRMT3 inhibition combined with immunotherapy on radiotherapy sensitization. (D) Patients with high PRMT3 and IDO1 expression had reduced CD8^{+} T cell infiltration. Scale bar: 100 μm. (E) IDO1 knockout LLC cells were generated. (F) PRMT3 expression in patient cohorts positively correlated with TFAP2A expression. Scale bar: 100 μm. (G-I) Constructs of PRMT3 enzyme-inactivated mutants, TFAP2A, and

TFAP2A-R363K mutants were established. Data represent the mean ± SD. $*P < 0.05$, $**P < 0.01$, $***P < 0.001$ and $****P < 0.0001$. Differences were tested using 1-way ANOVA test (C).
