# Supplementary material for: PRMT3 Drives IDO1-Dependent Radioresistance and Immunosuppression by Promoting Kynurenine Metabolism in Non–Small Cell Lung Cancer
Source: Cancer Res. 2025 Oct 23;86(2):421–37. doi: 10.1158/0008-5472.CAN-24-4162 (PMC12809119; doi:10.1158/0008-5472.CAN-24-4162)
Supplement: Supplementary Table S1 — The details of the detection antibodies involved in the study. [file can-24-4162_supplementary_table_s1_suppst1.pdf]

Supplementary Table S1. The details of the detection antibodies involved in the study.

Anti-body	Manufacturer	Catalogue numbers	Concentration	Application
Anti-PRMT3 antibody	Abcam	ab191562	1:100	IHC
IDO1 Polyclonal antibody	Proteintech	13268-1-AP	1:100	IHC
CD3 Monoclonal antibody	Proteintech	60181-1-Ig	1:1000	IHC
CD8a Monoclonal antibody	Proteintech	66868-1-Ig	1:6400	IHC
PRMT3 Polyclonal antibody	Proteintech	17628-1-AP	1:200	IHC
Ki67 Polyclonal antibody	Proteintech	28074-1-AP	1:2000	IHC
TFAP2A, AP-2 Monoclonal antibody	Proteintech	67076-1-Ig	1:300	IHC
HRP, Goat Anti-Rabbit IgG	Abbkine	A21020	1:500	IHC
HRP, Goat Anti-Mouse IgG	Abbkine	A21010	1:500	IHC
Anti-PRMT3 antibody	Abcam	ab191562	1:10000	WB
GAPDH Monoclonal antibody	Proteintech	60004-1-Ig	1:50000	WB
Phospho-Histone H2A.X (Ser139) (20E3) Rabbit mAb	Cell Signaling Technology	#9718	1:1000	WB
Anti-Indoleamine 2, 3-dioxygenase antibody	Abcam	ab211017	1:1000	WB
Anti-Transcription factor AP-2-alpha antibody	Abcam	ab108311	1:1000	WB
Anti-Flag tag Mouse mAb	PTM BIO	PTM-6075	1:5000	WB
Asymmetric Di-Methyl Arginine Motif [adme-R] MultiMab® Rabbit mAb	Cell Signaling Technology	#13522	1:1000	WB
Lamin B1	Proteintech	66095-1-Ig	1:20000	WB

Monoclonal antibody				
Beta Actin Polyclonal antibody	Proteintech	20536-1-AP	1:4000	WB
AffiniPure Goat Anti-Rabbit IgG (H+L)	Boster	BA1039	1:2000	WB
AffiniPure Goat Anti-Mouse IgG (H+L)	Boster	BA1038	1:2000	WB
HRP-conjugated Mouse anti-Rabbit IgG Light Chain	ABclonal	AS061	1:5000	WB
Anti-PRMT3 antibody	Abcam	ab191562	1:40	Co-IP
Rabbit Control IgG	ABclonal	AC005	1:100	Co-IP
Anti-Transcription factor AP-2-alpha antibody	Abcam	ab108311	1:20	Co-IP
Anti-Flag tag Mouse mAb	PTM BIO	PTM-6075	1:25	Co-IP
Anti-PRMT3 antibody	Abcam	ab191562	1:50	IF
Anti-Transcription factor AP-2-alpha antibody	Abcam	ab108311	1:50	IF
Phospho-Histone H2A.X (Ser139) (20E3) Rabbit mAb	Cell Signaling Technology	#9718	1:1000	IF
CY3 Conjugated AffiniPure Goat Anti-rabbit IgG (H+L)	Boster	BA1032	1:100	IF
