# Supplementary material for: PRMT3 Drives IDO1-Dependent Radioresistance and Immunosuppression by Promoting Kynurenine Metabolism in Non–Small Cell Lung Cancer
Source: Cancer Res. 2025 Oct 23;86(2):421–37. doi: 10.1158/0008-5472.CAN-24-4162 (PMC12809119; doi:10.1158/0008-5472.CAN-24-4162)
Supplement: Supplementary Table S2 — The lists of primers used for qRT-PCR or CHIP-qPCR. [file can-24-4162_supplementary_table_s2_suppst2.pdf]

Supplementary Table S2. The lists of primers used for qRT-PCR or CHIP-qPCR.

Gene	Primer Sequences
**PRMT1 (human)**	
Forward	5′- TGCGGTGAAGATCGTCAAAGCC -3′
Reverse	5′- GGACTCGTAGAAGAGGCAGTAG -3′
**PRMT2 (human)**	
Forward	5′- GCAGTTGGACATGAGAACCGTG -3′
Reverse	5′- AGGCTCTGGAAGTGGACGCTAA -3′
**PRMT3 (human)**	
Forward	5′- CACTGTCTGCTGAAGCCGCATT -3′
Reverse	5′- GTAGATGACGAGCAGGTTCTGAC -3′
**PRMT4 (CARM1; human)**	
Forward	5′- TTCCAGTCACCACTGTTCGCCA -3′
Reverse	5′- CCAGGAGGTTACTGGACTTGGA -3′
**PRMT5 (human)**	
Forward	5′- CTAGACCGAGTACCAGAAGAGG -3′
Reverse	5′- CAGCATACAGCTTTATCCGCCG -3′
**PRMT6 (human)**	
Forward	5′- TGGCTTTGCCATCTGGTTCCAG -3′
Reverse	5′- TAGAGGAGCGCCTGTTTCCAGT -3′
**PRMT7 (human)**	
Forward	5′- CCACGATGACTACTGCGTATGG -3′
Reverse	5′- GACGTACCGATCAGTTCTGTCC -3′
**PRMT8 (human)**	
Forward	5′- AGCAAGTGGTGACCAATGCCTG -3′
Reverse	5′- TGGACGTAGTCGTTGCGCTGTA -3′
**PRMT9 (human)**	
Forward	5′- GGACATTGGAGCAGGAACTGGA -3′
Reverse	5′- GTTTGCTGCCACGACATCACAG -3′
**GAPDH (human)**	
Forward	5′- CATGTTCGTCATGGGTGTGAA -3′
Reverse	5′- GGCATGGACTGTGGTCATGAG -3′
**IDO1 (human)**	
Forward	5′- CCAAAGGAACTGGAGGCACT -3′
Reverse	5′- GTGGCTCTGTTACAATGGGT -3′
**TFAP2A (human)**	
Forward	5′- GTGGCTGTCTTTACCCAGGC -3′
Reverse	5′- TGCAAGGAGCAATTGCCAAG -3′
**IDO1 (human; CHIP-qPCR)**	
Forward	5′- AGTAAAATGTTCTTCTCCGGCCA -3′
Reverse	5′- ACTGCCATATAGGAAGCCAGA -3′
**PD-1 (PDCD1; mouse)**	
Forward	5′- CGGTTTCAAGGCATGGTCATTGG -3′

Reverse	5′- TCAGAGTGTCGTCCTTGCTTCC -3′
**CYP1A1 (mouse)**	
Forward	5′- CATCACAGACAGCCTCATTGAGC -3′
Reverse	5′- CTCCACGAGATAGCAGTTGTGAC -3′
**CYP1B1 (mouse)**	
Forward	5′- TGTGCCTGCCACTATTACGG -3′
Reverse	5′- ACAACCTGGTCCAACTCAGC -3′
**GAPDH (mouse)**	
Forward	5′- CATCACTGCCACCCAGAAGACTG -3′
Reverse	5′- ATGCCAGTGAGCTTCCCGTTCAG -3′
