# Supplementary material for: PRMT3 Drives IDO1-Dependent Radioresistance and Immunosuppression by Promoting Kynurenine Metabolism in Non–Small Cell Lung Cancer
Source: Cancer Res. 2025 Oct 23;86(2):421–37. doi: 10.1158/0008-5472.CAN-24-4162 (PMC12809119; doi:10.1158/0008-5472.CAN-24-4162)
Supplement: Supplementary Table S3 — The sequences of shRNAs. [file can-24-4162_supplementary_table_s3_suppst3.pdf]

Supplementary Table S3. The sequences of shRNAs.

shRNA Targets	Sequences
shPRMT3-shRNA#1 sense (human)	5′- CCTTGGGAGAAAGAAGAGTAT -3′
shPRMT3-shRNA#2 sense (human)	5′- GCTGGCTACTTTGATATATAT -3′
shPRMT3-shRNA#3 sense (human)	5′- CCTTGTGGTATTAAGCATATA -3′
IDO1-shRNA#1 sense (human)	5′- CCATCTGCAAATCGTGACTAA -3′
IDO1-shRNA#2 sense (human)	5′- GCCCTTCAAGTGTTTCACCAA -3′
IDO1-shRNA#3 sense (human)	5′- CGTAAGGTCTTGCCAAGAAAT -3′
TFAP2A-shRNA#1 sense (human)	5′- GCTGAATTTCTCAACCGACAA -3′
TFAP2A-shRNA#2 sense (human)	5′- GCAAGATCCTTACTCCCACGT -3′
TFAP2A-shRNA#3 sense (human)	5′- GGAGACCTCTCGATCCACTCC -3′
IDO1-shRNA#1 sense (mouse)	5′- CCTCGCAATAGTAGATACTTA -3′
IDO1-shRNA#2 sense (mouse)	5′- CGTCTCTCTATTGGTGGAAAT -3′
