# Supplementary material for: PRMT3 Drives IDO1-Dependent Radioresistance and Immunosuppression by Promoting Kynurenine Metabolism in Non–Small Cell Lung Cancer
Source: Cancer Res. 2025 Oct 23;86(2):421–37. doi: 10.1158/0008-5472.CAN-24-4162 (PMC12809119; doi:10.1158/0008-5472.CAN-24-4162)
Supplement: Supplementary Table S4 — The antibody panels for sorting T cells by flow cytometry. [file can-24-4162_supplementary_table_s4_suppst4.pdf]

Supplementary Table S4. The antibody panels for sorting T cells by flow cytometry.

Channel	Panel	Catalogue No.	Company
APC/Cyanine7	CD45	#103115	Biolegend
FITC	CD3	#100203	Biolegend
PE/Cyanine7	CD4	#100421	Biolegend
PE	CD8a	#100707	Biolegend
APC	Granzyme B	#372203	Biolegend
PE/Cyanine7	CD3	#100219	Biolegend
PE/Cyanine7	CD45	#157205	Biolegend
APC	CD8a	#162305	Biolegend
PE	Granzyme B	#396405	Biolegend
