# Supplementary material for: PRMT3 Drives IDO1-Dependent Radioresistance and Immunosuppression by Promoting Kynurenine Metabolism in Non–Small Cell Lung Cancer
Source: Cancer Res. 2025 Oct 23;86(2):421–37. doi: 10.1158/0008-5472.CAN-24-4162 (PMC12809119; doi:10.1158/0008-5472.CAN-24-4162)
Supplement: Supplementary Table S5 — The sequence of wild type and mutant type of IDO1 in luciferase reporter assay. [file can-24-4162_supplementary_table_s5_suppst5.pdf]

Supplementary Table S5. The sequence of wild type and mutant type of IDO1 in luciferase reporter assay.

**IDO1**	
Wild type	GTTTAGGACTGCAGCCTTCATTTTCATTCAAAGATTTAAAAG TTTCCATAAAGTAAAATGTTCTTCTCCGGCCACCTGTTTTCAT AGTTCTGTGTTTTCCTTCAGGCCTTTCTGGCTTCCTATATGGC AGTAAGAAAATGATGTGCTTAATGATTACAAATTTCATATGGA ATACGAACTTTCAGTTTGTACATATGATG
Mutant type	GTTTAGGACTGCAGCCTTCATTTTCATTCAAAGATTTAAAAG TTTCCATAAAGTAAAATGTTCTTCTCCGGCCACCTGTTTTCAT AGTTCTGTGTTCCTTCCTGAATCTTTCTGGCTTCCTATATGGC AGTAAGAAAATGATGTGCTTAATGATTACAAATTTCATATGGA ATACGAACTTTCAGTTTGTACATATGATG
