# Supplementary material for: PRMT3 Drives IDO1-Dependent Radioresistance and Immunosuppression by Promoting Kynurenine Metabolism in Non–Small Cell Lung Cancer
Source: Cancer Res. 2025 Oct 23;86(2):421–37. doi: 10.1158/0008-5472.CAN-24-4162 (PMC12809119; doi:10.1158/0008-5472.CAN-24-4162)
Supplement: Supplementary Table S6 — The antibodies utilized in multiplex immunofluorescence assays. [file can-24-4162_supplementary_table_s6_suppst6.pdf]

Supplementary Table S6. The antibodies utilized in multiplex immunofluorescence assays.

Anti-body	Manufacturer	Catalogue numbers	Concentration
CD3ε (E4T1B) XP® Rabbit mAb	Cell Signaling Technology	#78588	1:400
Anti-CD4 antibody	Abcam	ab133616	1:200
Anti-CD8 alpha antibody	Abcam	ab217344	1:500
