# Supplementary material for: PRMT3 Drives IDO1-Dependent Radioresistance and Immunosuppression by Promoting Kynurenine Metabolism in Non–Small Cell Lung Cancer
Source: Cancer Res. 2025 Oct 23;86(2):421–37. doi: 10.1158/0008-5472.CAN-24-4162 (PMC12809119; doi:10.1158/0008-5472.CAN-24-4162)
Supplement: Supplementary Table S7 — Clinical characteristics of NSCLC patients for qPCR of PRMT3. [file can-24-4162_supplementary_table_s7_suppst7.pdf]

**Supplementary Table S7.** Clinical characteristics of NSCLC patients for qPCR of PRMT3.

| **Characteristic** | **PRMT3 expression** | | ***P value*** |
|---|---|---|---|
| | **Low (n=6)** | **High (n =6)** | |
| **Gender** | | | >0.9999 |
| Male | 5 (41.7%) | 5 (41.7%) | |
| Female | 1 (8.3%) | 1 (8.3%) | |
| **Age** | | | 0.558 |
| ≤60 | 3 (25.0%) | 4 (33.3%) | |
| >60 | 3 (25.0%) | 2 (16.7%) | |
| **Smoking Status** | | | 0.558 |
| Never-smokers | 3 (25.0%) | 2 (16.7%) | |
| Current/ex-smokers | 3 (25.0%) | 4 (33.3%) | |
| **AJCC stage** | | | - |
| III | 6(50.0%) | 6 (50.0%) | |
| **Histological types** | | | 0.221 |
| SCC | 5 (41.7%) | 3 (25.0%) | |
| ADC | 1 (8.3%) | 3 (25.0%) | |
| **RT efficacy** | | | 0.0005 |
| Response | 6 (50.0%) | 0 (0.0%) | |
| NO-Response | 0 (0.0%) | 6 (50.0%) | |

ADC: adenocarcinoma; SCC: squamous cell carcinoma

Response: CR+PR; NO-Response: SD+PD
