# Supplementary material for: PRMT3 Drives IDO1-Dependent Radioresistance and Immunosuppression by Promoting Kynurenine Metabolism in Non–Small Cell Lung Cancer
Source: Cancer Res. 2025 Oct 23;86(2):421–37. doi: 10.1158/0008-5472.CAN-24-4162 (PMC12809119; doi:10.1158/0008-5472.CAN-24-4162)
Supplement: Supplementary Table S8 — Expression differences of PRMT family genes in samples from the GSE25814 dataset before and after radiotherapy. [file can-24-4162_supplementary_table_s8_suppst8.pdf]

Supplementary Table S8. Expression differences of PRMT family genes in samples from the GSE25814 dataset before and after radiotherapy.

**ID**	**adj.*P*.Val**	***P*.Value**	**logFC**	**Gene.symbol**	**Gene.title**
7977820	0.2527	0.00770575	-0.3480875	PRMT5	protein arginine methyltransferase 5
7938890	0.2862	0.01031341	-0.5032373	PRMT3	protein arginine methyltransferase 3
8030437	0.6231	0.09560978	-0.2027107	PRMT1	protein arginine methyltransferase 1
7996785	0.8107	0.31628764	-0.1680618	PRMT7	protein arginine methyltransferase 7
8069450	0.8161	0.33020816	0.1277636	PRMT2	protein arginine methyltransferase 2
8103079	0.8752	0.48145659	-0.0666616	PRMT9	protein arginine methyltransferase 9
7903457	0.9494	0.73193394	0.0519796	PRMT6	protein arginine methyltransferase 6
7953181	0.9764	0.876799	-0.0258518	PRMT8	protein arginine methyltransferase 8
8025766	0.9536	0.75754378	-0.0382909	PRMT4	protein arginine methyltransferase 4
