# Supplementary material for: PRMT3 Drives IDO1-Dependent Radioresistance and Immunosuppression by Promoting Kynurenine Metabolism in Non–Small Cell Lung Cancer
Source: Cancer Res. 2025 Oct 23;86(2):421–37. doi: 10.1158/0008-5472.CAN-24-4162 (PMC12809119; doi:10.1158/0008-5472.CAN-24-4162)
Supplement: Supplementary Table S9 — Clinical characteristics of NSCLC patients for the IDO-PRMT3 correlation. [file can-24-4162_supplementary_table_s9_suppst9.pdf]

Supplementary Table S9. Clinical characteristics of NSCLC patients for the IDO-PRMT3 correlation (Fig 3L).

**Characteristic**	**PRMT3 expression**		***P* value**
	**Low (n=57)**	**High (n =56)**	
**Gender**			0.618
Male	45 (39.8%)	42 (37.2%)	
Female	12 (10.6%)	14 (12.4%)	
**Age**			0.774
≤60	29 (25.7%)	30 (26.5%)	
>60	28 (24.8%)	26 (23.0%)	
**Smoking Status**			0.486
Never-smokers	24 (21.2%)	20 (17.7%)	
Current/ex-smokers	33 (29.2%)	36 (31.9%)	
**T stage**			0.500
T1	5 (4.4%)	6 (5.3%)	
T2	25 (22.1%)	27 (23.9%)	
T3	17 (15.0%)	10 (8.9%)	
T4	10 (8.9%)	13 (11.5%)	
**N stage**			0.291
N0	1 (0.9%)	3 (2.7%)	
N1	6 (5.3%)	4 (3.5%)	
N2	24 (21.2%)	31 (27.4%)	
N3	26 (23.0%)	18 (16.0%)	
**M stage**			0.633
M0	55 (48.7%)	53 (46.9%)	
M1	2 (1.7%)	3 (2.7%)	
**AJCC stage**			0.633
III	55 (48.7%)	53 (46.9%)	
IV	2 (1.7%)	3 (2.7%)	
**Histological types**			0.371
SCC	43 (38.1%)	38 (33.6%)	
ADC	14 (12.4%)	18 (15.9%)	
