# Supplementary material for: PRMT3 Drives IDO1-Dependent Radioresistance and Immunosuppression by Promoting Kynurenine Metabolism in Non–Small Cell Lung Cancer
Source: Cancer Res. 2025 Oct 23;86(2):421–37. doi: 10.1158/0008-5472.CAN-24-4162 (PMC12809119; doi:10.1158/0008-5472.CAN-24-4162)
Supplement: Supplementary Table S10 — Clinical characteristics of NSCLC patients for survival data. [file can-24-4162_supplementary_table_s10_suppst10.pdf]

Supplementary Table S10. Clinical characteristics of NSCLC patients for survival data (Fig 8E).

Characteristic	PRMT3 expression		*P value*
	PRMT3(low) IDO1(low) (n=31)	PRMT3(high) IDO1(high) (n=31)	
**Gender**			0.534
Male	26 (42.9%)	23 (37.1%)	
Female	5 (8.1%)	8 (12.9%)	
**Age**			0.605
≤60	17 (27.4%)	20 (32.3%)	
>60	14 (22.6%)	11 (17.7%)	
**Smoking Status**			0.797
Never-smokers	12 (19.4%)	14 (22.6%)	
Current/ex-smokers	19 (30.6%)	17 (27.4%)	
**T stage**			0.178
T1	2 (3.2%)	5 (8.1%)	
T2	12 (19.4%)	17 (27.4%)	
T3	7 (11.3%)	5 (8.1%)	
T4	10 (16.1%)	4 (6.4%)	
**N stage**			0.299
N1	3 (4.8%)	4 (6.4%)	
N2	18 (29.1%)	12 (19.4%)	
N3	10 (16.1%)	15 (24.2%)	
**M stage**			-
M0	31(50.0%)	31 (50.0%)	
**AJCC stage**			-
III	31 (50.0%)	31(50.0%)	
**Histological types**			0.592
SCC	19 (30.6%)	22 (35.5%)	
ADC	12 (19.4%)	9 (14.5%)	
